# Corporate genome screening India (CoGsI) identified genetic variants association with T2D in young Indian professionals

**DOI:** 10.1038/s41598-024-84160-2

**Published:** 2025-01-02

**Authors:** Shah Fahad Husami, Tavleen Kaur, Love Gupta, Garima Rastogi, Lakhvinder Singh, Pooja Meena, Indu Sharma, Hemender Singh, Varun Sharma

**Affiliations:** 1 Unlock Wellness Pvt. Ltd., New Delhi, 110025 India; 2 NMC Genetics India Pvt. Ltd., Gurugram, Haryana 122001 India; 3 Pharmacology Divisions, CSIR-Indian Institute of Integrative Medicine, Jammu, 180001 India; 4 Keck School of Medicine, University of Southern California, Los Angeles, CA USA

**Keywords:** Type 2 diabetes, Genetics, Young professionals, SNPs, Genotyping, Genetics, Molecular biology, Diseases

## Abstract

Rising cases of type 2 diabetes (T2D) in India, especially in metropolitan cities is an increasing concern. The individuals that were most affected are young professionals working in the corporate sector. However, the corporate sector has remained the least explored for T2D risk predisposition. Considering corporate employees’ lifestyles and the role of gene-environment interaction in T2D susceptibility, the study aims to find genetic variants associated with T2D predisposition. In this first kind of study, 680 young professionals (284 T2D cases, and 396 controls) were diagnosed and screened for 2658 variants on an array designed explicitly for the CoGsI study. The variant filtering was done at Bonferroni p-value of 0.000028. The genetic data was analysed using PLINK v1.09, SPSS, R programming, VEP tool, and FUMA GWAS tool. Interestingly, 42 variants were associated with the T2D risk. Out of 42, three missense variants (rs1402467, rs6050, and rs713598) in Sulfotransferase family 1 C member 4 (*SULT1C4*), Fibrinogen Alpha Chain (*FGA*), and Taste 2 Receptor Member 38 (*TAS2R38*) and two untranslated region (UTR) variants (rs1063320 and rs6296) in Major Histocompatibility Complex, Class I, G (*HLA-G*) and 5-Hydroxytryptamine Receptor 1B (*HTR1B*) were associated with the T2D risk. CoGsI identified potential genomic markers increasing susceptibility to the early onset of T2D. Present findings provide insights into mechanisms underlying T2D manifestation in corporate professionals due to genetics interacting with occupational stress and urban lifestyles.

**Supplementary Information:**

The online version contains supplementary material available at 10.1038/s41598-024-84160-2.

## Background

Type 2 diabetes (T2D) is a pervasive^1^ and consequential health concern characterised by chronic glycemia due to insulin resistance, impaired insulin production, and sometimes both^2,3^. The World Health Organization reported 422 million global diabetes cases, projected to reach 700 million by 2045^4^.

T2D poses a significant burden, triggering complications like cardiovascular disorders, diabetic retinopathy, nephropathy, neuropathy, and many more, condensing quality of life^5,6^. The International Diabetes Federation estimated global healthcare expenditure related to diabetes to be over 825 billion USD by 2030 and 845 billion USD by 2045, constituting approximately 10% of total global healthcare spending^7^. To decrease such global burdens, it is crucial to decipher the factors affecting the severity of T2D^8^. It is a fact that genetics^9^, epigenetics^10^, and environmental factors play crucial roles in T2D progression^11,12^; mainly depending on the complex interaction of genetic variants and environmental factors^13^. Understanding the genetic and environmental contributors to T2D is vital, particularly in India, where it has a substantial impact^14^. Genetic risk variants across the human genome affect glucose metabolism, insulin signaling, and pancreatic function^15^. However, genetics operates within the context of environmental influences^16^, necessitating a comprehensive approach to address this global health challenge.

India, with a population exceeding 1.3 billion, faces a rapidly escalating T2D crisis, ranking second globally in the number of affected individuals^17^. Approximately 8.8% of India’s adult population, around 77 million individuals, have diabetes in 2023, with an alarming 44% being undiagnosed^18,19^. Diabetes-related healthcare expenditure accounted for about 10% of India’s total healthcare spending in 2023^20^. Moreover, studies have indicated the association of chronic stress with the elevated risk of T2D^21^. A large sample sized based T2D study in the Indian population has shown the increased risk of T2D in young North Indians, with a prevalence of 14.4%, having higher Indian diabetes risk score compared to the other parts of India, suggesting that the youth of India is at high risk of T2D specifically the young North Indians due to increased stress, improper sleep, lifestyle habits, sedentary lifestyle, more screen time, smoking, alcohol consumption, and others^22^. This rise in the cases of youth onset T2D in India and globally has become a major health concern. Multi-level strategies need to be developed among young individuals to prevent the pathogenesis of T2D, including identification of the exposures, social determinants, individual and family level characteristics, disorders associated with the increased risk of T2D, and many more^23^. Thus, it is pertinent to understand the genetic factors contributing to T2D among Indian professionals to address this epidemic and improve the health outcomes of the Indian population.

The risk of developing T2D depends not only on genetic factors but also on environmental factors. This can be explained as the interplay of the genes and gene-environment interaction. The genetic predisposition to T2D may result from how an individual is exposed to a specific environmental factor. For example, those who adopt an unhealthy diet based on their preferences for taste and food, low daily activity level, exposure to pollutants, poor gut microbiota, psychological and work-related stress, and many more, are highly susceptible to T2D^24–26^.

Our research team developed the idea of Corporate Genome Screening India (CoGsI), an innovative genetic research approach that leverages genetic data to investigate T2D genetic impact in young working professionals. In CoGsI, we conducted a screening among young Indian professionals to assess their susceptibility towards T2D as corporate sector jobs often come with heavy workloads and stress, deskbound work with minimal physical activity, and unhealthy eating habits. These factors are considered significant risk factors for predisposition to T2D. This strategy utilises the genetic diversity often found among employees or members of such organisations, enhancing the statistical power of genetic analyses and enabling a deeper understanding of genetics interplay with shared environmental factors unique to the group. Advanced genomic techniques, like array-based genotyping are needed to be employed in the corporate genome screening to identify genetic risk variants and shed light on complex interactions between genetics, lifestyle, and the environment. The variants incorporated in the array (2658) were previously being reported to be associated with various phenotypes of T2D and its associated complexities, including lifestyle-related conditions, neurological disorders, and behavioural and stress-related disorders. By examining these variants, we aimed to gain insights into how genetic variants with established roles in T2D and its associated complexities influence T2D risk among young professionals. This approach opens avenues for personalised healthcare strategies based on individuals’ unique genetic profiles.

## Materials and methods

### Ethical clearance

The study was initiated after seeking ethical approval from the institutional ethics review board (IERB) of NMC Genetics India Pvt. Ltd. notification vide number NMC/IERB/2023/07, dated: 22-04-2023. All experimental protocols were conducted according to the guidelines and regulations set by the IERB.

### Subjects

The present study is the first Corporate Genome Screening of young Indian professionals. The screening included 284 T2D (222 males and 62 females) individuals with age ranges between 24 and 50 years and 396 controls (304 males and 92 females) in the age group of 25 to 55 years. The post-hoc power of study calculation was performed using the Genetic Association Study (GAS) Power Calculator (https://csg.sph.umich.edu/abecasis/cats/gas_power_calculator/index.html)^27^. Further, all the cases were clinically diagnosed in accordance with the recommendations set by the American Diabetes Association^28^, and several clinical parameters were recorded, including Vitamin D3, Cholesterol levels, Liver profile, Protein, HbA1c, and many others that were evaluated in all the T2D affected individuals (Table 1).

**Table 1:** Distribution of the clinical parameters observed in cases and controls.

S No.	Blood parameters	Cases (*N* = 284)		Controls (*N* = 396)		*p*-value
		Mean	SD	Mean	SD	
	Age (in years)	38.98	7.62	40.07	12.19	0.2291
1.	25-OH VITAMIN D (TOTAL) (ng/mL)	22.44	15.34	29.72	18.76	3.42E−06*
2.	VITAMIN B-12 (pg/mL)	309.94	203.18	–	–	–
3.	APOLIPOPROTEIN - A1 (APO-A1) (mg/dL)	118.79	15.25	121.72	21.94	0.211
4.	APOLIPOPROTEIN - B (APO-B) (mg/dL)	97.49	21.05	90.78	19.38	0.018*
5.	APO B / APO A1 RATIO (APO B/A1) (Ratio)	0.83	0.21	0.799	0.32	0.007*
6.	TOTAL CHOLESTEROL (mg/dL)	185.37	37.07	181.88	42.17	0.324
7.	HDL CHOLESTEROL - DIRECT (mg/dL)	42.24	8.81	45.38	12.16	0.0009*
8.	HDL / LDL RATIO	0.38	0.14	0.42	0.28	0.042*
9.	LDL CHOLESTEROL - DIRECT (mg/dL)	120.48	30.44	121.66	36.12	0.692
10.	TRIGLYCERIDES (mg/dL)	146.30	87.49	142.99	84.72	0.666
11.	TC/ HDL CHOLESTEROL RATIO	5.07	8.54	4.2	1.24	0.111
12.	LDL / HDL RATIO	2.96	0.90	2.84	1.07	0.173
13.	VLDL CHOLESTEROL (mg/dL)	29.52	17.49	29.06	17.43	0.769
14.	NON-HDL CHOLESTEROL (mg/dL)	142.28	36.80	136.63	40.91	0.103
15.	ALKALINE PHOSPHATASE (U/L)	82.67	25.94	83.67	28.56	0.681
16.	BILIRUBIN - TOTAL (mg/dL)	0.73	0.32	0.68	0.34	0.090
17.	BILIRUBIN -DIRECT (mg/dL)	0.15	0.09	0.17	0.1	0.018*
18.	BILIRUBIN (INDIRECT) (mg/dL)	0.57	0.26	0.51	0.27	0.011*
19.	GAMMA GLUTAMYL TRANSFERASE (GGT) PHOTOMETRY (U/L)	35.99	56.91	36.04	42.97	0.991
20.	SGOT / SGPT RATIO CALCULATED	0.94	0.37	0.95	0.4	0.771
21.	ASPARTATE AMINOTRANSFERASE (SGOT ) PHOTOMETRY (U/L)	28.86	12.23	29.33	13.72	0.685
22.	PROTEIN - TOTAL PHOTOMETRY (g/dL)	7.23	0.49	7.08	0.41	0.0002*
23.	ALBUMIN - SERUM PHOTOMETRY (gm/dL)	4.35	0.36	4.3	0.31	0.096
24.	SERUM ALB/GLOBULIN RATIO CALCULATED	1.53	0.23	1.58	0.29	0.032*
25.	SERUM GLOBULIN (gm/dL)	2.89	0.40	2.76	0.37	0.002*
26.	HbA1c - (HPLC) (%)	7.93	3.56	5.98	1.34	1.27E−14*

SD: Standard deviation, N = Number of samples, * variables that have significant p-values < 0.05.

### Sample collection

The blood samples for the biochemical test were initially drawn to diagnose the subjects as cases or controls in the present study. Additionally, the 2mL blood sample was collected in EDTA collection tubes from cases and controls after seeking informed consent from all the subjects for the isolation of DNA. The blood samples were stored at -20° C until further processing.

### DNA isolation

The blood samples were processed further to isolate the genomic DNA. The DNA was isolated using the Qiagen DNA mini kit as per the manufacturer’s protocol. The qualitative and quantitative analysis of the DNA samples was performed using the agarose gel electrophoresis and Nanodrop (ThermoFisher) (Supplementary Fig. 1a,b,c).

### Genotyping

The DNA samples of cases and controls were genotyped using an array designed exclusively for the CoGsI screening (Supplementary Figs. 2, 3, 4, 5). The samples were screened for a total of 2658 variants. The detailed information about the sample genotyping was adopted from our previous studies^29,30^.

### Statistical analysis

**Fig. 1:**
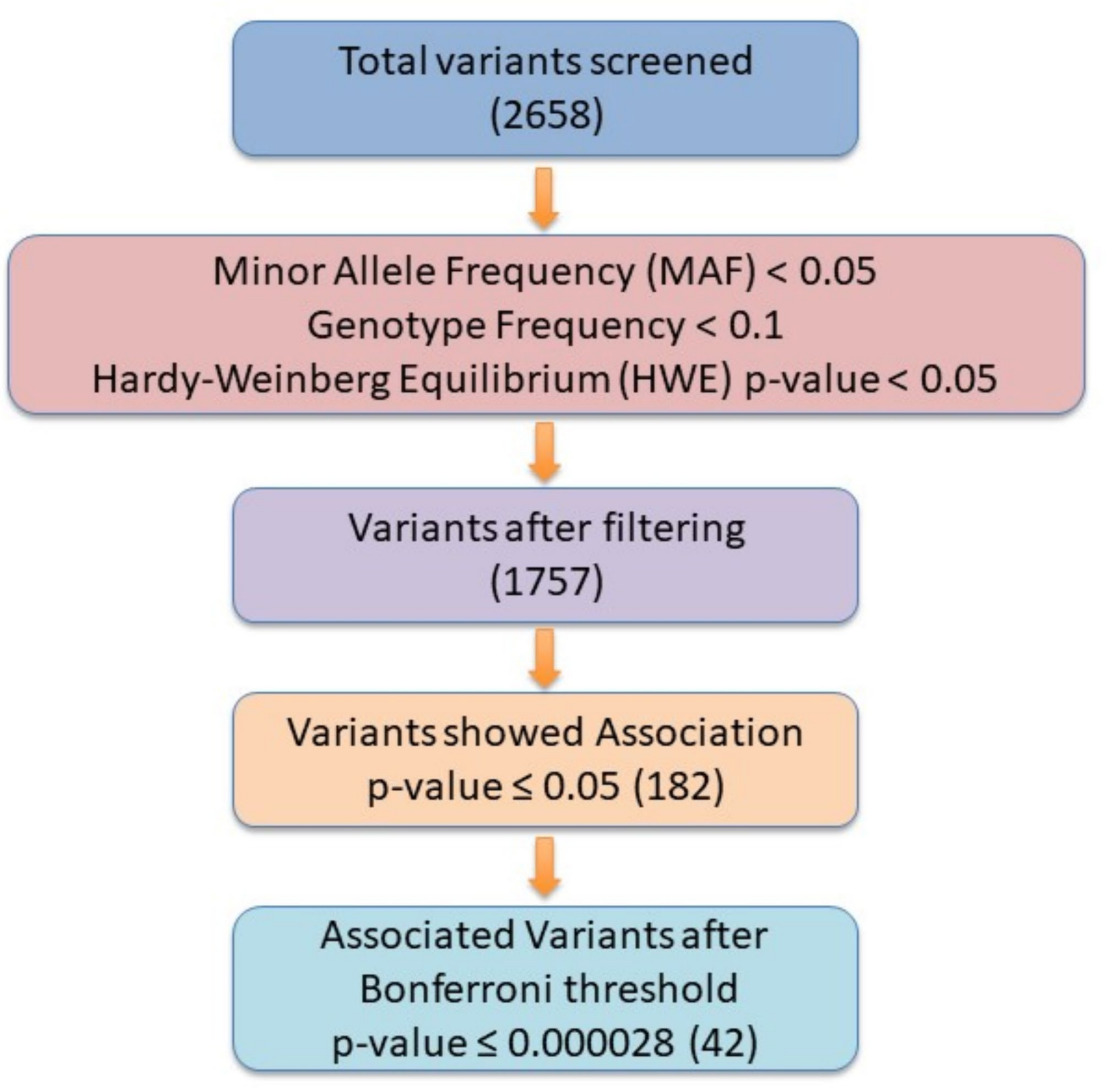
Flowchart depicting the variant filtering criteria used in the present study. The variant filtering criteria in the flowchart comprised of the minor allele frequency filter of < 0.05, genotype frequency of < 0.1, and Hardy Weinberg equilibrium of < 0.05. The association of the variants with T2D was observed at the Bonferroni threshold of ≤ 0.000028.

The statistical analysis of the genotyping data was conducted using the PLINK v1.09^31^. The association of the clinical parameters among cases and controls was performed using the SPSS v27 (IBM) and R programming. To ensure the robustness of our analysis, we applied stringent data filtration criteria, including a minor allele frequency threshold of less than 0.05, to exclude rare variants that may not provide sufficient statistical power for association tests based on the sample size of the present study, genotype frequency of less than 0.1 was considered to filter out variants with low homozygosity, and adherence to Hardy-Weinberg Equilibrium (HWE) with a significance level of less than 0.05 using PLINK v1.09^31^. Following these quality control measures, we observed that 1757 Single Nucleotide Polymorphisms (SNPs) remained for further analysis. We evaluated the association of these SNPs with T2D using comprehensive statistical assessments. Our investigation revealed a noteworthy outcome, with 182 genetic variants demonstrating significant associations with T2D (p-value < 0.05). To ensure the reliability of our findings, we rigorously corrected potential artifacts using the Bonferroni correction method (p-value ≤ 0.000028). The p-values of the association result have been adjusted with the confounding factors (age, gender, and BMI). To evaluate the correlation between the associated genetic variants with the physical activity level, occupational stress, and unhealthy diet, the Pearson correlation coefficient method was performed using Python script. Consequently, at the culmination of our analysis, we identified and confirmed 42 genetic variants after Bonferroni correction that maintain a robust and statistically significant association with the development of T2D. The variant filter criterion is represented in Fig. 1.

### Functional annotation of associated variants/genes

The annotation of the variants for their functional role was evaluated using the Variant Effect Predictor tool (Ensembl)^32^. The associated genes were further annotated using the GENE2FUNC tool of the FUMA GWAS tool^33^. The gene expression was evaluated using the GTEx data for 54 tissue sets. In addition, the Gene ontology analysis for the role of genes in the biological processes was determined using the MSigDb data from the FUMA GWAS tool. The network analysis of the associated genes was performed using the GeneMANIA tool (https://genemania.org/)^34^.

## Results

### Clinical parameters

The cases and controls belong to the age group of 38 (± 7.62) and 40 (± 12.19) years, respectively. Eleven of 26 blood parameters were significantly associated with T2D in cases with p-values of < 0.05 (Table 1). The levels of vitamin D3 in cases and controls are 22.44 (± 15.34) and 29.72 (± 18.76) ng/ml, respectively, which falls in the insufficiency and normal range of vitamin D3 levels recommended for better health. All the individuals have Apolipoprotein B in the normal range < 110 mg/dL. The individuals have a moderate risk related to the Apo B/ ApoA1 ratio because they fall in the moderate range of 0.7–0.9. The HDL cholesterol levels of cases and controls were 42.24 (± 8.81) and 45.38 (± 12.16) mg/dL, which falls in the normal range. The levels of bilirubin in cases and controls were 0.15 (± 0.09) and 0.17 (± 0.1) for direct bilirubin and 0.57 (± 0.26) and 0.51 (± 0.27) for indirect bilirubin respectively, as compared to the normal range of 0.2–1.2 mg/dL. The total protein content for cases were 7.23 (± 0.49) and 7.08 (± 0.41) g/dL, respectively. Both groups fell within the 6–8.5 g/dL range for total protein. The normal range of serum albumin/ globulin ratio is 1–2, and the observed values for cases and controls were 1.53 ± 0.23 and 1.58 ± 0.29. Serum globulin levels in the cases and controls were 2.89 ± 0.40 and 2.76 ± 0.37 compared to the normal range of 2–3.5 g/dL. Regarding glycemic control, the average HbA1c level in cases was 7.93 (± 3.56), which falls in the diabetic range. On the other hand, for the controls, the HbA1c level was 5.98 ± 1.34, which falls in the normal range. This information summarises the key findings related to age, vitamin D3 levels, Apo-B, cardiovascular risk, HDL cholesterol, bilirubin, total protein, albumin/ globulin ratio, and diabetic levels in cases and controls.

The estimated power of the study was 96.3% (Supplementary Fig. 6), considering a p-value threshold of 0.000028, with 8% of the T2D prevalence^35^, 0.2 as minor allele frequency with an average odds ratio of 2. A power greater than 80%^36^; indicates very little chance of Type II errors, ensuring that the study is sensitive enough to detect even small effects and providing strong evidence of association with the selected sample size.

### Genotyping

**Fig. 2:**
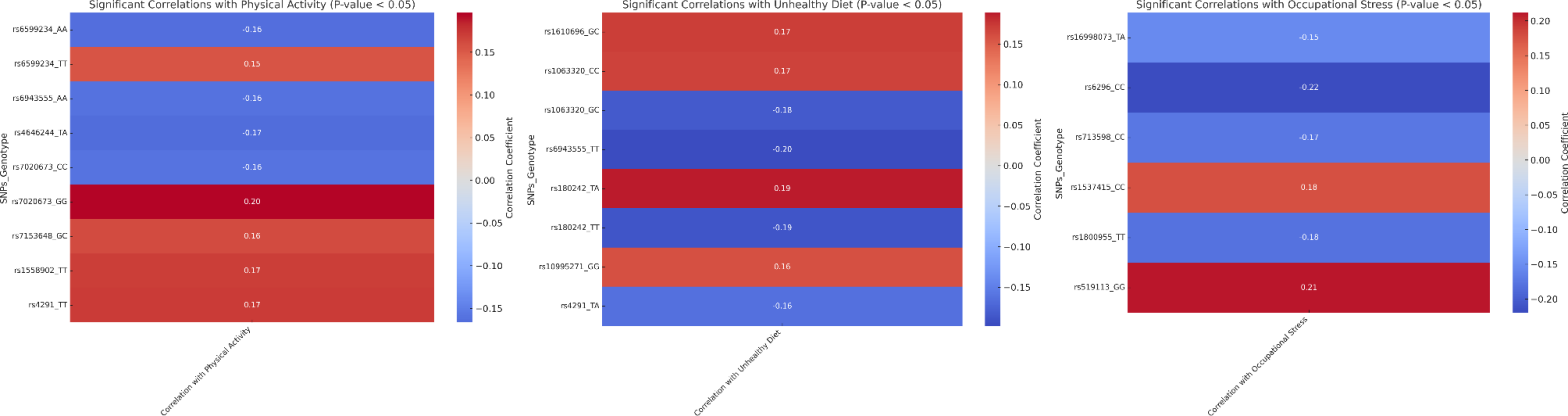
Correlations of significant variants and their genotypes with lifestyle/environmental factors including Physical Activity, Unhealthy Diet, and Occupational Stress in Young Indian Professionals.

From the total of 2658 variants following the quality control measures, the present study has revealed 42 genetic variants demonstrating significant associations with T2D. The 42 variants were found to provide a strong risk predisposition to T2D. All these variants were associated with T2D risk with higher odds at a very high significance level (Table 2). Moreover, the variants were further evaluated for their epistatic effect (supplementary Table 1) and their association under additive (supplementary Table 2), dominant (supplementary Table 3), and recessive model (supplementary Table 4). The epistatic analysis revealed that the variant of the Calcium Voltage-Gated Channel Subunit Alpha1 C (CACNA1C) gene showed interaction with the Interleukin 1 Beta (*IL1B)*, Fibrinogen Alpha Chain *(FGA)*, T Cell Immunoglobulin And Mucin Domain Containing 4 *(TIMD4)*, Major Histocompatibility Complex, Class I, G *(HLA-G)*, 5-Hydroxytryptamine Receptor 1B *(HTR1B)*, Poliovirus receptor-related 2 *(PVRL2)*, and Transmembrane Serine Protease 6 *(TMPRSS6).* The interaction analysis further depicted the elevated risk at higher significance levels with T2D in the population. Most of the associated variants were intronic, while three of them, viz. rs1402467, rs6050, and rs713598, were missense variants that would probably alter the structure of the protein. In addition, variants rs1063320 and rs6296 were 3´ UTR and 5´ UTR variants, respectively, that might play an essential role in regulating gene expression. In addition, the interactive analysis of the 42 associated genetic variants with occupational stress, unhealthy diet, and physical activity was evaluated to determine the complex mechanism behind the predisposition of T2D in young Indian professionals. It was found that some of the genetic variants were found to be significantly interacting with these environmental factors in causing the T2D susceptibility in the studied population (Fig. 2, Supplementary Table 5).

**Table 2:** Genetic variants found to be associated with T2D in studied cohort.

SNP	Gene	Func cons	Codon	Effect allele	EAF in cases	EAF in controls	Alternate allele	OR (95%CI)	*P*_adjusted_
rs12027135	*TMEM57*	Intron variant		T	0.6549	0.327	A	3.906 (3.11–4.906)	5.85E-33
rs10918594	*RP11-227F8.2*	NA		C	0.6092	0.4003	G	2.335 (1.873–2.911)	2.94E-14
rs61759167	*PRDM16*	Intron variant		T	0.3644	0.2538	C	1.686 (1.334–2.131)	1.13E-05
rs1143623	*IL1B*	2KB upstream variant		C	0.6761	0.3258	G	4.32 (3.432–5.437)	2.68E-37
rs1402467	*SULT1C4*	Missense variant	D (Asp) > E (Glu)	G	0.5458	0.3712	C	2.035 (1.635–2.534)	1.66E-10
rs6599234	*SCN5A*	NA		A	0.6831	0.3144	T	4.701 (3.728–5.927)	3.60E-41
rs12493607	*TGFBR2*	Intron variant		C	0.6496	0.3586	G	3.317 (2.647–4.155)	3.12E-26
rs4533622	*CTNNB1*	Intron variant		A	0.6127	0.4141	C	2.238 (1.795–2.789)	5.13E-13
rs16998073	*PRDM8*	NA		T	0.7042	0.3101	A	5.296 (4.187–6.699)	1.14E-46
rs6050	*FGA*	Missense variant	T [66] > A [67]	G	0.5616	0.3434	A	2.449 (1.963–3.056)	1.19E-15
rs10520514	*TENM3*	Intron variant		T	0.6004	0.404	A	2.216 (1.778–2.761)	9.10E-13
rs1501908	*TIMD4*	None		C	0.6849	0.3093	G	4.852 (3.846–6.122)	1.16E-42
rs12654264	*HMGCR*	Intron variant		A	0.5827	0.423	T	1.905 (1.531–2.37)	6.15E-09
rs1610696	*HLA-G*	500B Downstream variant		C	0.6831	0.3081	G	4.841 (3.837–6.108)	1.43E-42
rs1063320	*HLA-G*	3 Prime UTR variant		G	0.6585	0.3081	C	4.33 (3.441–5.449)	1.69E-37
rs6296	*HTR1B*	5 Prime UTR variant		C	0.6426	0.3573	G	3.234 (2.583–4.049)	2.80E-25
rs1801132	*ESR1*	Synonymous variant		G	0.6144	0.3939	C	2.452 (1.965–3.058)	1.03E-15
rs11755527	*BACH2*	Intron variant		G	0.5792	0.4304	C	1.822 (1.465–2.266)	6.24E-08
rs713598	*MGAM*	Missense variant	A [Ala] > P [66]	C	0.6725	0.3194	G	4.375 (3.476–5.508)	6.49E-38
rs6943555	*AUTS2*	Intron variant		A	0.6743	0.3258	T	4.285 (3.405–5.393)	6.00E-37
rs180242	*GNG11*	2KB upstream variant		A	0.6303	0.3813	T	2.766 (2.214–3.456)	1.30E-19
rs6968865	*LOC101927609*	Intron variant		T	0.5986	0.3939	A	2.294 (1.841–2.859)	9.30E-14
rs4646244	*NAT2*	Intron variant		A	0.6303	0.3396	T	3.314 (2.647–4.151)	2.86E-26
rs1537415	*GLT6D1*	Intron variant		G	0.7218	0.3157	C	5.626 (4.439–7.13)	1.95E-49
rs7020673	*GLIS3*	Intron variant		C	0.5475	0.428	G	1.617 (1.302–2.009)	1.35E-05
rs11014166	*CACNB2*	Intron variant		T	0.706	0.2879	A	5.94 (4.686–7.53)	1.61E-52
rs12772424	*TCF7L2*	Intron variant		T	0.6673	0.3093	A	4.477 (3.555–5.638)	5.35E-39
rs10995271	*ZNF365*	Intron variant		C	0.6127	0.3801	G	2.58 (2.068–3.22)	2.44E-17
rs187238	*IL18*	2KB upstream variant		C	0.7377	0.2348	G	9.162 (7.143–11.75)	1.26E-75
rs1800955	*DRD4*	2KB upstream variant		C	0.5792	0.4432	T	1.73 (1.391–2.15)	7.48E-07
rs10848653	*CACNA1C*	Intron variant		G	0.5	0.274	A	2.65 (2.112–3.324)	1.57E-17
rs7153648	*SIX1*	None		C	0.7306	0.2753	G	7.142 (5.605–9.099)	6.52E-62
rs684513	*CHRNA5*	Intron variant		G	0.6074	0.3712	C	2.621 (2.1-3.271)	7.52E-18
rs1558902	*FTO*	Intron variant		A	0.6655	0.3582	T	3.564 (2.84–4.472)	5.43E-29
rs3865188	*RP11-2L4.1*	None		A	0.6039	0.4078	T	2.214 (1.777–2.758)	9.85E-13
rs11869286	*STARD3*	Intron variant		G	0.6954	0.2992	C	5.347 (4.229–6.761)	2.62E-47
rs4291	*ACE*	2KB upstream variant		T	0.6281	0.3649	A	2.94 (2.35–3.678)	1.21E-21
rs519113	*PVRL2*	Intron variant		C	0.6831	0.2929	G	5.203 (4.118–6.573)	4.63E-46
rs11672691	*AC011526.1*	Intron variant		A	0.4542	0.3384	G	1.627 (1.304–2.03)	1.52E-05
rs1884613	*RP5-881L22.5*	Intron variant		G	0.6567	0.2929	C	4.617 (3.664–5.818)	1.95E-40
rs5756506	*TMPRSS6*	Intron variant		C	0.7332	0.2835	G	6.944 (5.451–8.847)	3.80E-60
rs713875	*RP3-438O4.4*	Intron variant		G	0.6232	0.3952	C	2.532 (2.028–3.159)	1.07E-16

SNP: Single Nucleotide Polymorphism, Func Cons: Functional consequence, EAF: Effect Allele Frequency (represents the frequency of the allele tested for association in the studied population), OR: Odds ratio, CI: Confidence Interval, p_adjusted_: Adjusted p- value for age, gender and BMI.

### Functional annotation

**Fig. 3:**
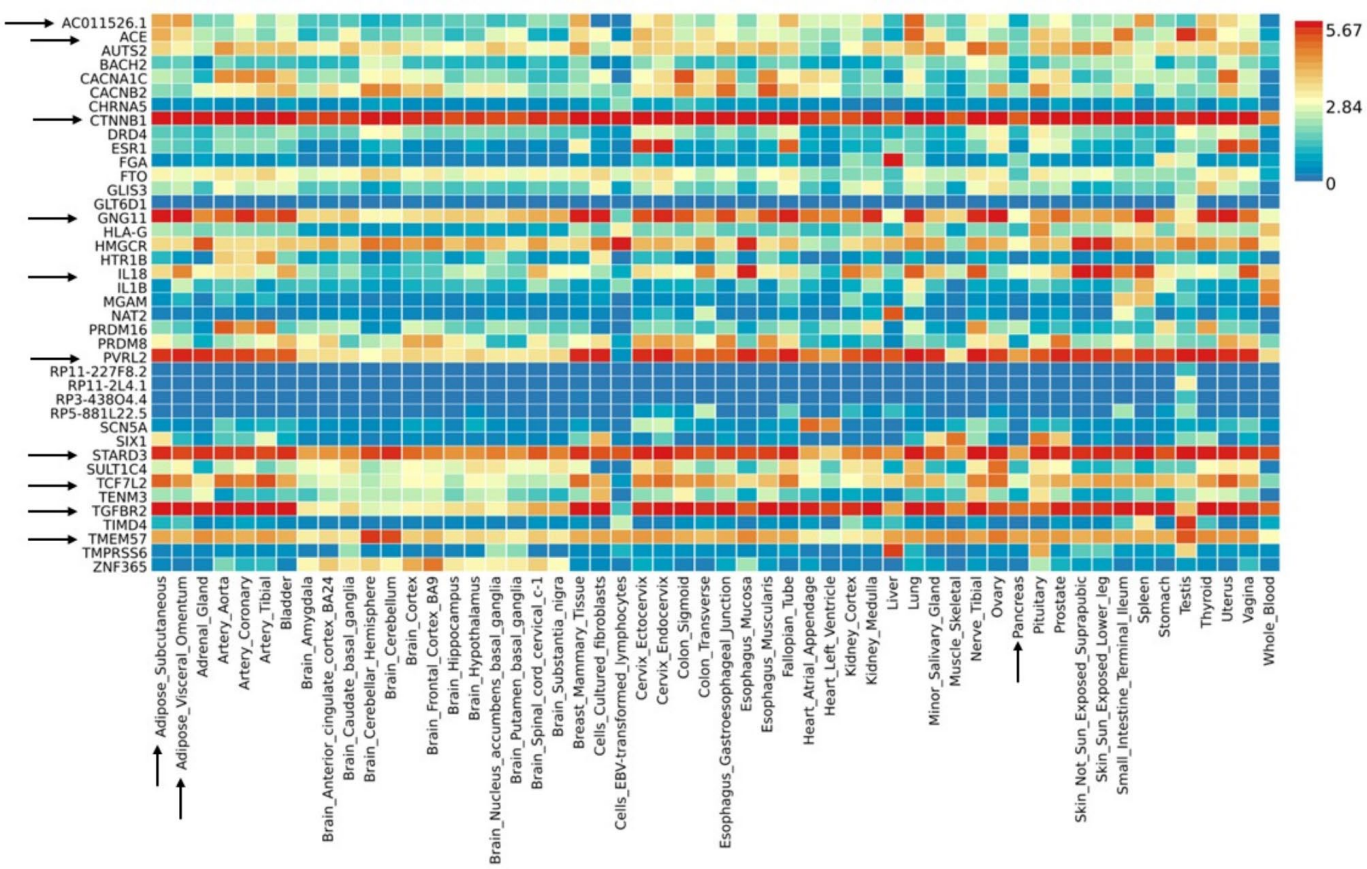
Heatmap representing the expression of the genes in 54 tissue sets data of GTEx database highlighting the possibly important genes and their expression in the specific tissues. The significantly associated genes of the present study were highlighted with the black arrow and their expression in the specific tissues including Adipose subcutaneous tissue, adipose visceral omentum tissue, and the pancreas tissue has been highlighted with a black arrow.

The genes of the associated variants were further annotated for their functional relevance. The heatmap was generated that depicts some interesting findings (Fig. 3). The genes have shown different expression profiles in the 54 tissues set. It was found that the genes including Angiotensin I Converting Enzyme (*ACE)*, Catenin Beta 1 *(CTNNB1)*, G Protein Subunit Gamma 11 *(GNG11)*, Interleukin 18 *(IL18)*,* PVRL2*, StAR Related Lipid Transfer Domain Containing 3 *(STARD3)*, Transcription Factor 7 Like 2 *(TCF7L2)*, Transforming Growth Factor Beta Receptor 2 *(TGFBR2)*, and Transmembrane Protein 57 (*TMEM57)* were up-regulated in the subcutaneous adipose tissue, visceral omentum and pancreas. In contrast, the rest of the other genes were comparatively under-expressed in other tissue sets.

Furthermore, the gene ontology (GO) analysis for the role of genes in the biological processes was performed using the MSigDB data. It was observed that PR/SET Domain 16 (*PRDM16)*,* TGFBR2*,* CTNNB1*,* HTR1B*, Estrogen Receptor 1 *(ESR1)*,* IL18*,* ACE*, *IL1B*, SIX Homeobox 1 *(SIX1)*, FTO Alpha-Ketoglutarate Dependent Dioxygenase *(FTO)*, 3-Hydroxy-3-Methylglutaryl-CoA Reductase *(HMGCR)*, Sodium Voltage-Gated Channel Alpha Subunit 5 *(SCN5A)*, Calcium Voltage-Gated Channel Auxiliary Subunit Beta 2 (*CACNB2)*, Dopamine Receptor D4 *(DRD4)*, and *CACNA1C* were significantly associated with the brown fat cell differentiation, response to lipid calcium ion transmembrane transport, negative regulation of glucose transmembrane transport, lipid localisation, MAPK cascade, regulation of calcium ion import, calcium ion homeostasis, calcium ion transmembrane transport, and regulation of voltage-gated calcium channel activity (Supplementary Table 6). These results suggest their potential role in glucose homeostasis, insulin transport, and the risk of developing obesity, which ultimately leads to the development of T2D. Similarly, the GO analysis of the GWAS catalog reported genes using the Gene2Func function of FUMA GWAS tool has shown a significant association of *PRDM16*,* TGFBR2*,* CTNNB1*,* PRDM8*,* ESR1*, GLIS Family Zinc Finger 3 *(GLIS3)*,* CACNB2*, Zinc Finger Protein 365 *(ZNF365)*,* TCF7L2*,* FTO*,* ACE*,* HMGCR*,* TIMD4*,* HLA-G*, N-Acetyltransferase 2 *(NAT2)*, and *CACNB2* with various risk factors of T2D including blood pressure, LDL cholesterol levels, Apolipoprotein B levels, triglyceride levels, circulating leptin levels, fasting insulin levels, total cholesterol levels, and blood glucose levels (Supplementary Table 7).

**Fig. 4:**
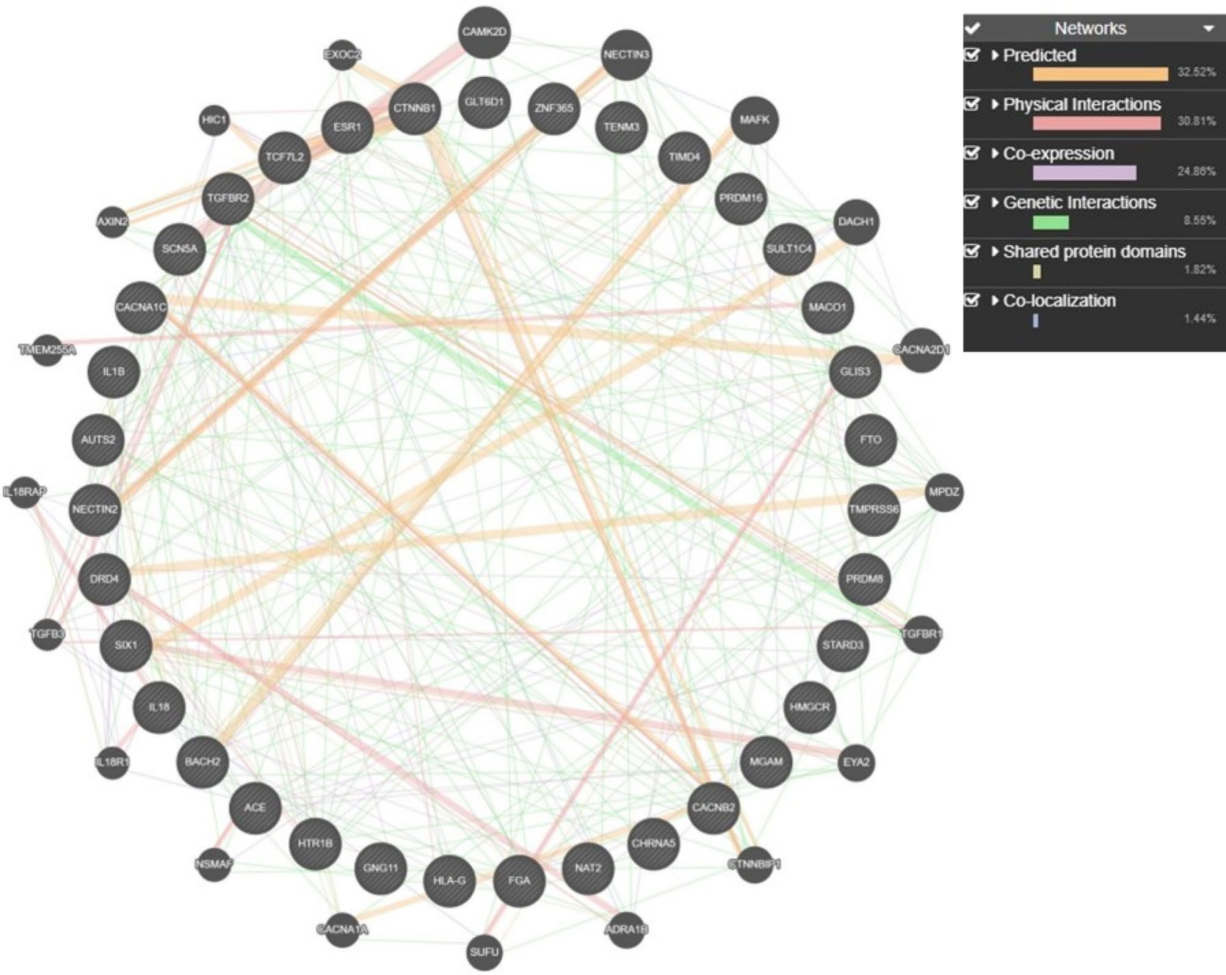
Network analysis depicting the gene-gene interaction of the associated genes depicting the genetic, physical, predicted interactions, shared protein domain, co-localisation, and co-expression. The different interactions have been shown in different colors as represented in the key provided in the figure. Majority of the interactions were found to predicted, physical and genetic in nature.

Moreover, the network analysis of the risk genes depicts the predicted interaction among the genes contributing about 32.52%, physical interaction of 30.81%, co-expression of 24.86%, genetic interaction of 8.55%, shared protein domains contributing about 1.82% and co-localisation of 1.44% (Fig. 4).

## Discussion

The present study is the first corporate-based genetic screening of T2D in India. In this study, the variants of the genes that were previously reported to be associated with various lifestyle complexities, neurological, stress, and behavioural disorders were screened in the young professionals of the corporate sector. India is considered the diabetic capital of the world^37^. Many young people, specifically those residing in metropolitan areas, suffer from T2D, which is probably due to environmental factors, including their lifestyle habits and deskbound jobs. No efforts have been made to conduct genetic screening in the corporate sector to evaluate predisposition to T2D until now.

The present study has found a significant association of the eleven clinical parameters with T2D risk in the current population screened, viz. 25-OH Vitamin D, Apolipoprotein - B (APO-B), (APO B/A1) (Ratio), HDL Cholesterol – Direct, HDL / LDL Ratio, Bilirubin -Direct (mg/dL), Bilirubin (Indirect) (mg/dL), Protein - Total photometry, Serum ALB/Globulin ratio, Serum Globulin, and HbA1c - (HPLC). All these factors are critical clinical biomarkers for the indication of pre-diabetic and diabetic conditions.

Moreover, the present study identified the genetic variants in the *TMEM57*, *RP11-227F8.2*, *PRDM16*, *IL1B*, *SULT1C4*, *SCN5A*, *TGFBR2*, *CTNNB1*, *PRDM8*, *FGA*, *TENM3*, *TIMD4*, *HMGCR*, *HLA-G*, *HTR1B*, *ESR1*, *BACH2*, *TAS2R38*, Activator Of Transcription And Developmental Regulator AUTS2 *(AUTS2)*,* GNG11*,* LOC101927609*,* NAT2*,* GLT6D1*,* GLIS3*,* CACNB2*,* TCF7L2*,* ZNF365*,* IL18*,* DRD4*,* CACNA1C*,* SIX1*,* CHRNA5*,* FTO*,* RP11-2L4.1*,* STARD3*,* ACE*,* PVRL2*,* AC011526.1*,* RP5-881L22.5*,* TMPRSS6*, and *RP3-438O4.4* associated with risk of T2D. Interestingly, these candidate genes/loci were associated with various complex disorders/traits including diabetes, cardiovascular disease, cancer, dyslipidemia, taste, and many more^38–43^ where environmental factors contribute significantly to their pathogenesis.

Captivatingly, three missense variants (rs1402467, rs6050, and rs713598) in *SULT1C4*,* FGA*, and *TAS2R38* were associated with the risk of T2D in the studied population. *SULT1C4* is a sulfotransferase, and studies have found increased protein expression in individuals with fatty liver and obesity^44^, indicating its substantial role in T2D pathogenesis. Studies have also shown the association of the FGA gene with different types of diabetes, including T2D, type 1 diabetes, diabetic neuropathy, and gestational diabetes^45–47^. Similarly, the *TAS2R38*, which encodes a bitter taste receptor and is localised in the gustatory system, has been found to negatively regulate glucose homeostasis, which might lead to the risk of T2D^41,48,49^.

Moreover, two UTR variants (rs1063320 and rs6296) in *HLA-G* and *HTR1B* were associated with the risk of T2D in the studied population, and studies have also identified the association of *HLA-G* in the risk of gestational diabetes mellitus and type 1 diabetes^50,51^. The *HTR1B*, which is a negative regulator of serotonin, might act as a crucial factor for T2D pathogenesis^52^. It has been observed that serotonin levels have decreased in the brain in conditions like type 1 diabetes and T2D. The negative regulation of serotonin is linked to T2D through its impact on insulin secretion and glucose metabolism. The serotonin is vital for the control of metabolic processes including pancreatic beta cell insulin secretion. Studies indicate several ways in which serotonin might enhance glucose-stimulated insulin secretion (GSIS) via serotonylation, where serotonin links itself to proteins involved in insulin exocytosis. Serotonin pathways dysregulation, whether caused by environmental or hereditary elements, might compromise these metabolic processes, hence increasing the T2D risk^53,54^. Studies have also reported that increasing serotonin levels has an association with a positive effect on T2D condition^52,55,56^. The polymorphism in *HTR1B* was also associated with increased BMI in the African American ancestry^35^, a risk factor for T2D pathogenesis^57^. Similarly, other genes whose variants were found to be associated with the present study were also likely to have a role in glucose homeostasis, dyslipidemia, obesity, diabetic nephropathy, diabetic neuropathy, type 1 diabetes, and many more that are crucial risk factors for T2D susceptibility^39,58–65^.

The correlation analysis showed interesting findings where the GG genotype of rs7020673 exhibits a positive correlation (0.20) with less physical activity (Fig. 2), these findings corroborate with the association analyses where the recessive model was showing higher odds than other model i.e., 2.2 (1.5–3.1 at 95% CI, p-value 9.59E-06) (supplementary Table 4), implying that those carrying this genotype might be at greater risk to have T2D because of less exercise. Whereas, the TA genotype of rs4646244 showed a negative correlation (-0.17) (Fig. 2) with physical activity, potentially offering some protection against T2D by promoting higher activity levels. Furthermore, the rs1063320 showed a negative correlation (-0.18) (Fig. 2), indicating that GC genotype carriers with healthy eating habits are less likely to have T2D risk. On the other hand, the rs180242 TT genotype shows a positive correlation (0.19) with an unhealthy diet, implying a greater inclination for bad dietary practices that might increase the risk of T2D. Moreover, rs6296 CC genotype negative correlation (-0.22) with occupational stress (Fig. 2). On the other hand, the rs519113 GG genotype was positively correlated (0.21) with occupational stress (Fig. 2), thereby, possibly increasing T2D risk.

The GO analysis in the present study also indicates the role of associated genes with risk factors of T2D based on their role in regulating dyslipidemia, insulin transport, MAPK pathway, glucose homeostasis, and many more. The network analysis of the gene-gene interaction depicts the interplay of the genes. Most of the genes were found to interact with each other in terms of physical, co-expression, and genetic interaction. The key genes such as *TMEM57*,* PRDM16*,* IL1B*,* SCN5A*,* TGFBR2*,* CTNNB1*,* HMGCR*,* HTR1B*,* ESR1*, and *FTO* are observed to be interacting through various types of interactions. Predicted interactions show potential relationships that may not yet be experimentally confirmed but suggest implications for determining the similar functions of the interacting genes. Physical interactions indicate direct protein-protein interactions that play a crucial role in understanding the biochemical pathways. Similarly, co-expression of the genes expressed together suggests the involvement of the interacting genes in related biological processes. The genes that were found to show the genetic interactions highlight that their effect can be modified by another interacting gene, revealing their epistatic relationships. The shared protein domains hint at the functional similarities, while co-localisation highlights the proteins that share the same cellular compartment. These interactions provide insights into the genetic architecture of T2D, highlighting potential therapeutic targets and thereby guiding future research into the disease’s pathogenesis. Thus, the individual carrying variations in more than one of these genes would possibly be at a higher risk of developing T2D.

Moreover, the study provides valuable genetic and clinical information that is crucial for the development of personalised healthcare strategies and interventions that are specifically tailored to individuals’ distinct genetic profiles. This will ultimately aid in the prevention and management of T2D in the Indian population, particularly among young professionals who are dealing with the challenges of urban lifestyles and occupational stress.

## Conclusion

The present study is the first to investigate genetic predispositions to T2D among young corporate professionals in India. In this study, 42 genetic loci were found to be significantly associated with the T2D risk, including missense variants in *SULT1C4*,* FGA*, and *TAS2R38*, and UTR variants in *HLA-G* and *HTR1B*. These genes are involved in pathways regulating glucose homeostasis, insulin signaling, obesity, inflammation, and stress response. The study highlights the role of genetic factors interacting with occupational stress, physical activity, and unhealthy diet. The findings of this study will pave the way for personalised risk assessments and tailored prevention strategies, emphasising the importance of further exploring gene-environment interactions to address the rising T2D burden in India. The replication studies are warranted to validate these findings and expand genomic research in understudied populations.

## Electronic supplementary material

Below is the link to the electronic supplementary material.

Supplementary Material 1

## Data Availability

All data described in this study are provided within the article and Supplementary Material. The raw genotyping and clinical data are available from the corresponding authors upon request.

## Code availability

The code used in the manuscript is present in the public repository https://github.com/sharmavarun840.
