# Supplementary material for: Corporate genome screening India (CoGsI) identified genetic variants association with T2D in young Indian professionals
Source: Sci Rep. 2025 Jan 2;15:506. doi: 10.1038/s41598-024-84160-2 (PMC11697386; doi:10.1038/s41598-024-84160-2)
Supplement: Supplementary file 1 — Supplementary Material 1 [file 41598_2024_84160_MOESM1_ESM.docx]

**Supplementary Figures**


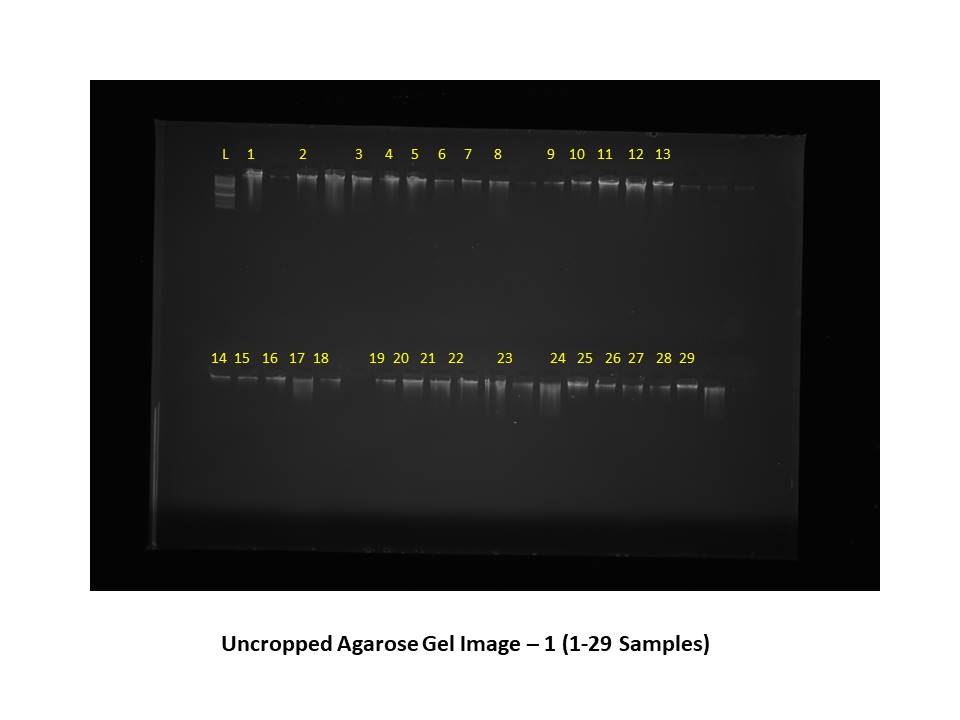


**Supplementary Figure 1a**: Representative example uncropped agarose gel images of DNA samples used in the present study


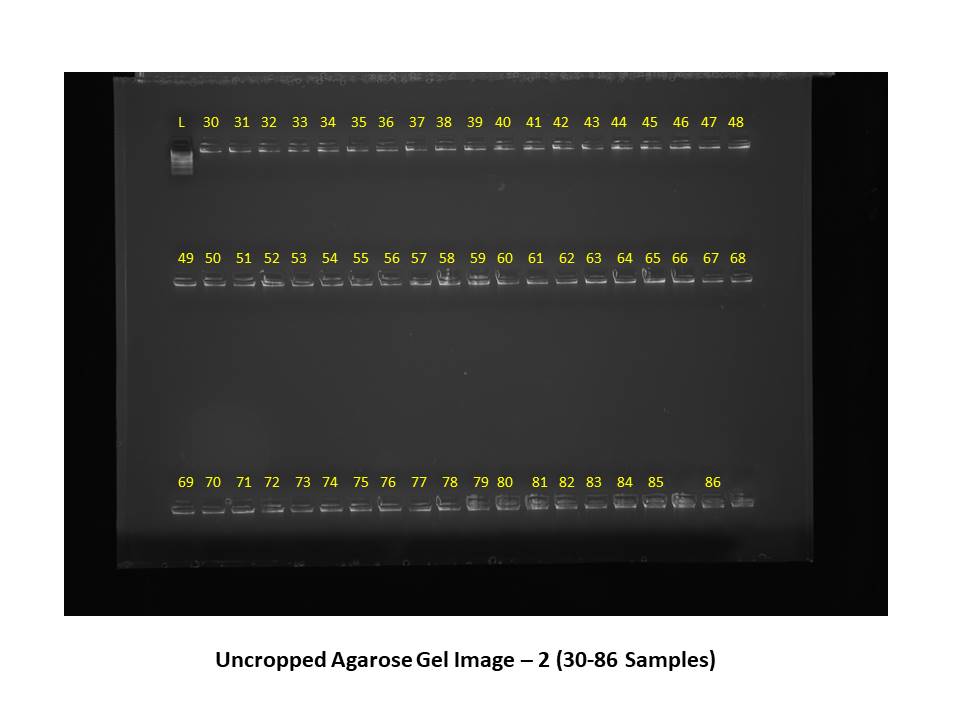


**Supplementary Figure 1b**: Representative example uncropped agarose gel images of DNA samples used in the present study


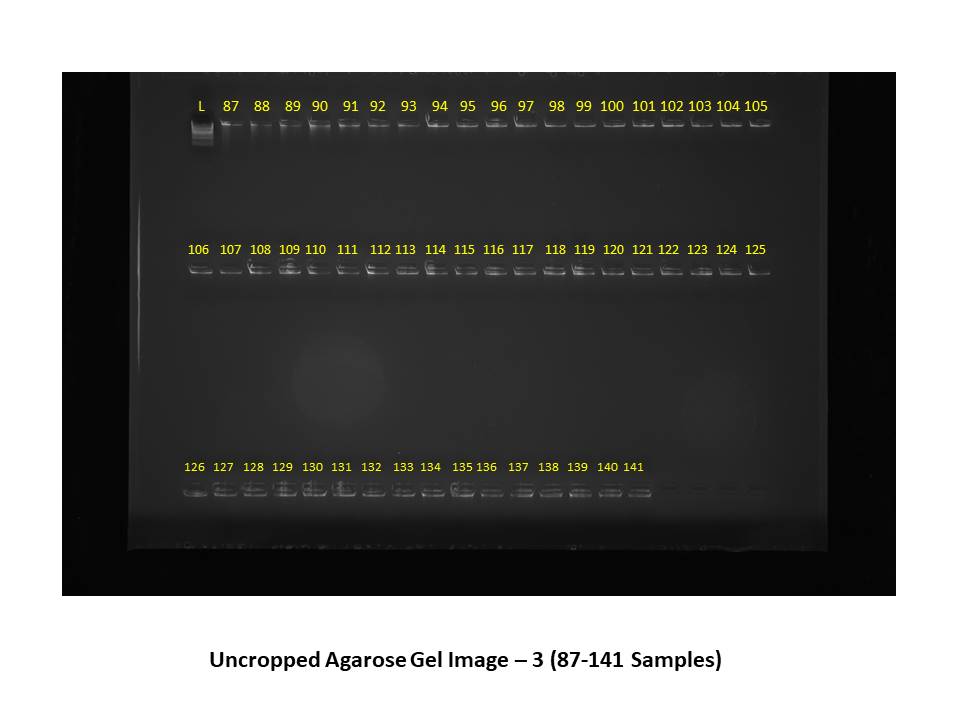


**Supplementary Figure 1c**: Representative example uncropped agarose gel images of DNA samples used in the present study

**Supplementary Figure 2**: Representative example genotyping plot of variant rs12042319 demonstrating the genotyping quality in the present study (blue and red color clusters depicts homozygous genotypes and purple color clusters depict heterozygous genotypes).

**Supplementary Figure 3**: Representative example genotyping plot of variant rs12947788 demonstrating the genotyping quality in the present study (blue and red color clusters depicts homozygous genotypes and purple color clusters depict heterozygous genotypes).

**Supplementary Figure 4**: Representative example genotyping plot of variant rs13149993 demonstrating the genotyping quality in the present study (blue and red color clusters depicts homozygous genotypes and purple color clusters depict heterozygous genotypes).

**Supplementary Figure 5**: Representative example genotyping plot of variant rs136161 demonstrating the genotyping quality in the present study (blue and red color clusters depicts homozygous genotypes and purple color clusters depict heterozygous genotypes).

**
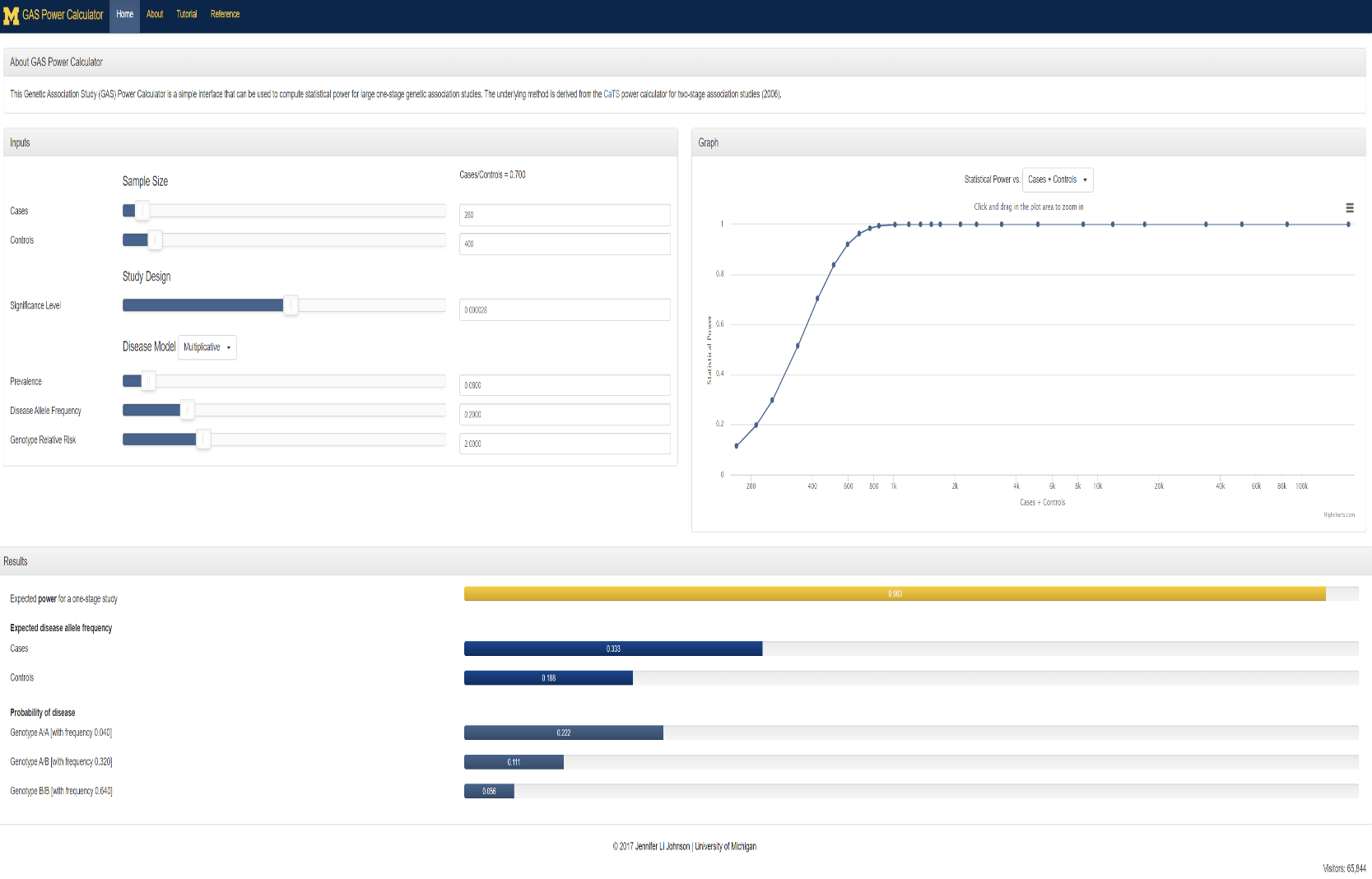
**

**Supplementary Figure 6:** The image depicting the input parameters for calculating the post-hoc power of study (96.3%) using the GAS power calculator.

**Supplementary Table:**

**Supplementary Table 1:** Epistasis analysis depicting the variants that showed an interactive effect on the risk of T2D in the studied population group.

| **S.No.** | **CHR1** | **SNP1** | **Gene** | **CHR2** | **SNP2** | **Gene** | **OR** | **p**_adjusted_ |
| --- | --- | --- | --- | --- | --- | --- | --- | --- |
| 1 | 2 | rs1143623 | *IL1B* | 12 | rs10848653 | *CACNA1C* | 4.214 | 5.77E-06 |
| 2 | 4 | rs6050 | *FGA* | 12 | rs10848653 | *CACNA1C* | 4.002 | 1.94E-05 |
| 3 | 5 | rs1501908 | *TIMD4* | 12 | rs10848653 | *CACNA1C* | 4.306 | 3.35E-06 |
| 4 | 6 | rs1610696 | *HLA-G* | 12 | rs10848653 | *CACNA1C* | 4.061 | 1.19E-05 |
| 5 | 6 | rs6296 | *HTR1B* | 12 | rs10848653 | *CACNA1C* | 3.929 | 1.38E-05 |
| 6 | 12 | rs10848653 | *CACNA1C* | 19 | rs519113 | *PVRL2* | 3.443 | 7.44E-05 |
| 7 | 12 | rs10848653 | *CACNA1C* | 22 | rs5756506 | *TMPRSS6* | 4.43 | 1.89E-05 |

CHR: Chromosome number, SNP: Single Nucleotide Polymorphism, OR: Odds ratio, p_adjusted_: Adjusted p-value

**Supplementary Table 2:** Association analysis of the risk variants in the additive model.

| **S.No** | **CHR** | **SNP** | **BP** | **A1** | **OR (95% CI)** | **p**_adjusted_ |
| --- | --- | --- | --- | --- | --- | --- |
| 1 | 1 | rs61759167 | 3175023 | T | 1.809 (1.43-2.29) | 8.09E-07 |
| 2 | 1 | rs12027135 | 25449242 | T | 4.111 (3.152-5.362) | 1.80E-25 |
| 3 | 1 | rs10918594 | 162060898 | C | 2.419 (1.909-3.065) | 2.57E-13 |
| 4 | 2 | rs1402467 | 108378352 | G | 2.056 (1.632-2.591) | 9.54E-10 |
| 5 | 2 | rs1143623 | 112838252 | C | 4.269 (3.287-5.544) | 1.36E-27 |
| 6 | 3 | rs12493607 | 30641447 | C | 3.442 (2.676-4.427) | 6.30E-22 |
| 7 | 3 | rs6599234 | 38673809 | A | 4.619 (3.541-6.025) | 1.62E-29 |
| 8 | 3 | rs4533622 | 41200847 | A | 1.858 (1.527-2.261) | 5.99E-10 |
| 9 | 4 | rs16998073 | 80263187 | T | 5.354 (4.066-7.05) | 6.65E-33 |
| 10 | 4 | rs6050 | 154586438 | G | 6.504 (3.543-11.94) | 1.52E-09 |
| 11 | 4 | rs10520514 | 181861392 | T | 2.204 (1.755-2.767) | 1.00E-11 |
| 12 | 5 | rs12654264 | 75352778 | A | 2 (1.582-2.527) | 6.64E-09 |
| 13 | 5 | rs1501908 | 156971158 | C | 4.983 (3.79-6.551) | 1.23E-30 |
| 14 | 6 | rs1063320 | 29830972 | G | 3.982 (3.108-5.101) | 7.84E-28 |
| 15 | 6 | rs1610696 | 29831026 | C | 4.404 (3.414-5.681) | 3.58E-30 |
| 16 | 6 | rs6296 | 77462543 | C | 2.921 (2.326-3.667) | 2.81E-20 |
| 17 | 6 | rs11755527 | 90248512 | G | 1.838 (1.467-2.302) | 1.19E-07 |
| 18 | 6 | rs1801132 | 151944387 | G | 2.399 (1.909-3.015) | 5.83E-14 |
| 19 | 7 | rs6968865 | 17247645 | T | 2.182 (1.75-2.72) | 3.98E-12 |
| 20 | 7 | rs6943555 | 70341037 | A | 4.046 (3.151-5.196) | 6.54E-28 |
| 21 | 7 | rs180242 | 93920284 | A | 2.888 (2.262-3.688) | 1.88E-17 |
| 22 | 7 | rs713598 | 141973545 | C | 4.013 (3.132-5.14) | 4.02E-28 |
| 23 | 8 | rs4646244 | 18390208 | A | 3.25 (2.551-4.141) | 1.40E-21 |
| 24 | 9 | rs7020673 | 4291747 | C | 1.577 (1.276-1.949) | 2.43E-05 |
| 25 | 9 | rs1537415 | 135637876 | G | 5.339 (4.06-7.021) | 4.02E-33 |
| 26 | 10 | rs11014166 | 18419869 | T | 5.74 (4.346-7.58) | 7.56E-35 |
| 27 | 10 | rs10995271 | 62678726 | C | 2.443 (1.951-3.058) | 6.64E-15 |
| 28 | 10 | rs12772424 | 113120792 | T | 4.863 (3.692-6.406) | 2.34E-29 |
| 29 | 11 | rs1800955 | 636784 | C | 8.623 (3.168-23.47) | 2.47E-05 |
| 30 | 11 | rs187238 | 112164265 | C | 9.887 (7.077-13.81) | 4.09E-41 |
| 31 | 12 | rs10848653 | 2378773 | G | 2.719 (1.193-6.199) | 0.01737 |
| 32 | 14 | rs7153648 | 60655808 | C | 6.986 (5.196-9.392) | 6.59E-38 |
| 33 | 15 | rs684513 | 78566058 | G | 2.609 (2.066-3.293) | 7.53E-16 |
| 34 | 16 | rs1558902 | 53769662 | A | 3.65 (2.833-4.701) | 1.22E-23 |
| 35 | 16 | rs3865188 | 82617112 | A | 2.123 (1.702-2.647) | 2.35E-11 |
| 36 | 17 | rs11869286 | 39657603 | G | 5.453 (4.135-7.192) | 3.17E-33 |
| 37 | 17 | rs4291 | 63476833 | T | 2.931 (2.307-3.725) | 1.40E-18 |
| 38 | 19 | rs11672691 | 41479679 | A | 1.675 (1.383-2.028) | 1.32E-07 |
| 39 | 19 | rs519113 | 44873027 | C | 5.332 (4.043-7.032) | 2.07E-32 |
| 40 | 20 | rs1884613 | 44351775 | G | 4.466 (3.449-5.782) | 6.96E-30 |
| 41 | 22 | rs713875 | 30196498 | G | 2.592 (2.04-3.293) | 6.41E-15 |
| 42 | 22 | rs5756506 | 37071352 | C | 7.687 (5.572-10.6) | 1.89E-35 |

CHR: Chromosome number, SNP: Single Nucleotide Polymorphism, BP: Base pair, A1: Minor allele, OR: Odds ratio, p_adjusted_: Adjusted p-value

**Supplementary Table 3:** Association analysis of the risk variants in the dominant model.

| **S.No** | **CHR** | **SNP** | **BP** | **A1** | **OR (95% CI)** | **p**_adjusted_ |
| --- | --- | --- | --- | --- | --- | --- |
| 1 | 1 | rs61759167 | 3175023 | T | 0.4803 (0.3327-0.6933) | 9.02E-05 |
| 2 | 1 | rs12027135 | 25449242 | T | 1.529 (1.08-2.165) | 0.01663 |
| 3 | 1 | rs10918594 | 162060898 | C | 1.054 (0.7646-1.452) | 0.7498 |
| 4 | 2 | rs1402467 | 108378352 | G | 1.153 (0.8403-1.581) | 0.3785 |
| 5 | 2 | rs1143623 | 112838252 | C | 1.295 (0.9137-1.835) | 0.1463 |
| 6 | 3 | rs12493607 | 30641447 | C | 1.079 (0.7714-1.51) | 0.6564 |
| 7 | 3 | rs6599234 | 38673809 | A | 1.287 (0.904-1.833) | 0.1615 |
| 8 | 3 | rs4533622 | 41200847 | A | 0.07622 (0.04709-0.1234) | 1.11E-25 |
| 9 | 4 | rs16998073 | 80263187 | T | 0.8198 (0.5708-1.178) | 0.2823 |
| 10 | 4 | rs6050 | 154586438 | G | 13.28 (7.011-25.15) | 2.08E-15 |
| 11 | 4 | rs10520514 | 181861392 | T | 0.9882 (0.7198-1.357) | 0.9415 |
| 12 | 5 | rs12654264 | 75352778 | A | 1.118 (0.8159-1.533) | 0.4869 |
| 13 | 5 | rs1501908 | 156971158 | C | 1.199 (0.8383-1.714) | 0.3206 |
| 14 | 6 | rs1063320 | 29830972 | G | 0.9876 (0.7011-1.391) | 0.9431 |
| 15 | 6 | rs1610696 | 29831026 | C | 1.086 (0.7657-1.54) | 0.644 |
| 16 | 6 | rs6296 | 77462543 | C | 0.7447 (0.5355-1.036) | 0.07986 |
| 17 | 6 | rs11755527 | 90248512 | G | 1.089 (0.7969-1.489) | 0.5922 |
| 18 | 6 | rs1801132 | 151944387 | G | 0.9279 (0.6738-1.278) | 0.6466 |
| 19 | 7 | rs6968865 | 17247645 | T | 0.8947 (0.6515-1.229) | 0.492 |
| 20 | 7 | rs6943555 | 70341037 | A | 0.789 (0.5593-1.113) | 0.177 |
| 21 | 7 | rs180242 | 93920284 | A | 1.093 (0.7878-1.518) | 0.5935 |
| 22 | 7 | rs713598 | 141973545 | C | 0.879 (0.6234-1.239) | 0.4618 |
| 23 | 8 | rs4646244 | 18390208 | A | 1.053 (0.7561-1.468) | 0.7584 |
| 24 | 9 | rs7020673 | 4291747 | C | 0.7599 (0.557-1.037) | 0.08306 |
| 25 | 9 | rs1537415 | 135637876 | G | 0.9555 (0.6637-1.376) | 0.8067 |
| 26 | 10 | rs11014166 | 18419869 | T | 1.048 (0.726-1.512) | 0.8037 |
| 27 | 10 | rs10995271 | 62678726 | C | 0.9217 (0.6687-1.27) | 0.6182 |
| 28 | 10 | rs12772424 | 113120792 | T | 1.191 (0.8348-1.7) | 0.3346 |
| 29 | 11 | rs1800955 | 636784 | C | 15.82 (5.713-43.81) | 1.08E-07 |
| 30 | 11 | rs187238 | 112164265 | C | 1 (0.6606-1.515) | 0.9989 |
| 31 | 12 | rs10848653 | 2378773 | G | 43.82 (18.79-102.2) | 2.13E-18 |
| 32 | 14 | rs7153648 | 60655808 | C | 1.014 (0.691-1.487) | 0.9444 |
| 33 | 15 | rs684513 | 78566058 | G | 0.9503 (0.6884-1.312) | 0.7563 |
| 34 | 16 | rs1558902 | 53769662 | A | 0.9076 (0.6467-1.274) | 0.5751 |
| 35 | 16 | rs3865188 | 82617112 | A | 0.8415 (0.6131-1.155) | 0.2856 |
| 36 | 17 | rs11869286 | 39657603 | G | 0.9075 (0.6316-1.304) | 0.5996 |
| 37 | 17 | rs4291 | 63476833 | T | 1.002 (0.7216-1.391) | 0.991 |
| 38 | 19 | rs11672691 | 41479679 | A | 0.000000000434 (0-inf) | 0.9911 |
| 39 | 19 | rs519113 | 44873027 | C | 1.134 (0.7905-1.627) | 0.4941 |
| 40 | 20 | rs1884613 | 44351775 | G | 0.7079 (0.4983-1.006) | 0.05382 |
| 41 | 22 | rs713875 | 30196498 | G | 1.33 (0.9615-1.841) | 0.08494 |
| 42 | 22 | rs5756506 | 37071352 | C | 1.379 (0.9246-2.057) | 0.1152 |

CHR: Chromosome number, SNP: Single Nucleotide Polymorphism, BP: Base pair, A1: Minor allele, OR: Odds ratio, p_adjusted_: Adjusted p-value

**Supplementary Table 4:** Association analysis of the risk variants in the recessive model.

| **S.No** | **CHR** | **SNP** | **BP** | **A1** | **OR (95% CI)** | **p**_adjusted_ |
| --- | --- | --- | --- | --- | --- | --- |
| 1 | 1 | rs61759167 | 3175023 | T | 3.444 (2.186-5.424) | 9.58E-08 |
| 2 | 1 | rs12027135 | 25449242 | T | 4.862 (3.312-7.137) | 6.80E-16 |
| 3 | 1 | rs10918594 | 162060898 | C | 3.078 (2.137-4.434) | 1.57E-09 |
| 4 | 2 | rs1402467 | 108378352 | G | 2.431 (1.655-3.572) | 5.98E-06 |
| 5 | 2 | rs1143623 | 112838252 | C | 5.865 (4.003-8.591) | 1.08E-19 |
| 6 | 3 | rs12493607 | 30641447 | C | 4.848 (3.318-7.083) | 3.35E-16 |
| 7 | 3 | rs6599234 | 38673809 | A | 6.584 (4.465-9.709) | 1.87E-21 |
| 8 | 3 | rs4533622 | 41200847 | A | 7.257 (5.069-10.39) | 2.62E-27 |
| 9 | 4 | rs16998073 | 80263187 | T | 10.77 (7.064-16.41) | 2.22E-28 |
| 10 | 4 | rs6050 | 154586438 | G | 0.9997 (0.6389-1.564) | 0.9989 |
| 11 | 4 | rs10520514 | 181861392 | T | 2.898 (2.023-4.151) | 6.59E-09 |
| 12 | 5 | rs12654264 | 75352778 | A | 2.278 (1.585-3.274) | 8.59E-06 |
| 13 | 5 | rs1501908 | 156971158 | C | 7.513 (5.028-11.23) | 7.51E-23 |
| 14 | 6 | rs1063320 | 29830972 | G | 6.818 (4.587-10.13) | 2.22E-21 |
| 15 | 6 | rs1610696 | 29831026 | C | 7.248 (4.905-10.71) | 2.71E-23 |
| 16 | 6 | rs6296 | 77462543 | C | 5.31 (3.661-7.702) | 1.38E-18 |
| 17 | 6 | rs11755527 | 90248512 | G | 2.112 (1.482-3.009) | 3.47E-05 |
| 18 | 6 | rs1801132 | 151944387 | G | 3.406 (2.373-4.887) | 2.94E-11 |
| 19 | 7 | rs6968865 | 17247645 | T | 3.125 (2.185-4.47) | 4.39E-10 |
| 20 | 7 | rs6943555 | 70341037 | A | 7.845 (5.267-11.69) | 3.95E-24 |
| 21 | 7 | rs180242 | 93920284 | A | 3.782 (2.611-5.476) | 1.91E-12 |
| 22 | 7 | rs713598 | 141973545 | C | 7.334 (4.95-10.86) | 2.92E-23 |
| 23 | 8 | rs4646244 | 18390208 | A | 4.791 (3.263-7.034) | 1.30E-15 |
| 24 | 9 | rs7020673 | 4291747 | C | 2.224 (1.561-3.169) | 9.59E-06 |
| 25 | 9 | rs1537415 | 135637876 | G | 9.692 (6.507-14.44) | 5.62E-29 |
| 26 | 10 | rs11014166 | 18419869 | T | 10.31 (6.792-15.66) | 6.74E-28 |
| 27 | 10 | rs10995271 | 62678726 | C | 3.576 (2.49-5.136) | 5.26E-12 |
| 28 | 10 | rs12772424 | 113120792 | T | 7.203 (4.77-10.88) | 5.99E-21 |
| 29 | 11 | rs1800955 | 636784 | C | 0.8552 (0.5703-1.282) | 0.4492 |
| 30 | 11 | rs187238 | 112164265 | C | 21.96 (13.23-36.44) | 6.53E-33 |
| 31 | 12 | rs10848653 | 2378773 | G | 0.1403 (0.04223-0.4662) | 0.001348 |
| 32 | 14 | rs7153648 | 60655808 | C | 13.56 (8.78-20.94) | 6.36E-32 |
| 33 | 15 | rs684513 | 78566058 | G | 3.772 (2.594-5.484) | 3.68E-12 |
| 34 | 16 | rs1558902 | 53769662 | A | 5.905 (4.021-8.67) | 1.31E-19 |
| 35 | 16 | rs3865188 | 82617112 | A | 3.104 (2.177-4.427) | 3.99E-10 |
| 36 | 17 | rs11869286 | 39657603 | G | 10.4 (6.793-15.91) | 4.14E-27 |
| 37 | 17 | rs4291 | 63476833 | T | 4.226 (2.903-6.15) | 5.25E-14 |
| 38 | 19 | rs11672691 | 41479679 | A | 5.271 (3.641-7.631) | 1.32E-18 |
| 39 | 19 | rs519113 | 44873027 | C | 8.707 (5.731-13.23) | 3.58E-24 |
| 40 | 20 | rs1884613 | 44351775 | G | 9.939 (6.435-15.35) | 3.97E-25 |
| 41 | 22 | rs713875 | 30196498 | G | 2.88 (2.017-4.114) | 5.99E-09 |
| 42 | 22 | rs5756506 | 37071352 | C | 11.48 (7.526-17.5) | 8.66E-30 |

CHR: Chromosome number, SNP: Single Nucleotide Polymorphism, BP: Base pair, A1: Minor allele, OR: Odds ratio, p_adjusted_: Adjusted p-value

**Supplementary Table 5:** Pearson correlation coefficient between the genetic variations and the environmental factors like physical activity, unhealthy food, and occupational stress.

| **SNPs_Genotype** | **Correlation with Physical Activity** | **P-value for Physical Activity** | **Correlation with Unhealthy Food** | **P-value for Unhealthy Food** | **Correlation with Occupational Stress** | **P-value for Occupational Stress** |
| --- | --- | --- | --- | --- | --- | --- |
| **rs12027135_AA** | -0.0987961 | 0.203996426 | 0.079932218 | 0.304496989 | 0.011749276 | 0.880213247 |
| **rs12027135_TA** | 0.114595854 | 0.140308213 | -0.01512095 | 0.846215736 | -0.02246765 | 0.773192383 |
| **rs12027135_TT** | -0.03357152 | 0.666684636 | -0.10782391 | 0.165449906 | 0.019369766 | 0.803781786 |
| **rs10918594_CC** | 0.075786412 | 0.330344097 | -0.0741547 | 0.340891986 | 0.060095674 | 0.440427318 |
| **rs10918594_GC** | -0.02899256 | 0.709944506 | -0.05303272 | 0.496083196 | -0.09979416 | 0.199441601 |
| **rs10918594_GG** | -0.02868541 | 0.712881674 | 0.112053665 | 0.149381162 | 0.056496606 | 0.468333741 |
| **rs61759167_CC** | -0.12651484 | 0.103269555 | 0.069166454 | 0.374444822 | -0.05107623 | 0.51213247 |
| **rs61759167_TC** | 0.045987578 | 0.555097365 | -0.00978312 | 0.900144199 | 0.096647355 | 0.214056179 |
| **rs61759167_TT** | 0.097478011 | 0.210126083 | -0.06831096 | 0.380395994 | -0.03700204 | 0.634970256 |
| **rs1143623_CC** | 0.012111278 | 0.87655143 | -0.01845464 | 0.812877547 | 0.055528418 | 0.476003106 |
| **rs1143623_GC** | 0.009637868 | 0.901619248 | -0.11888356 | 0.125965537 | -0.10505868 | 0.17663059 |
| **rs1143623_GG** | -0.01587746 | 0.838624144 | 0.128121033 | 0.098930582 | 0.076064167 | 0.328569684 |
| **rs1402467_CC** | 0.07718022 | 0.321501728 | 0.043806291 | 0.57403424 | 0.000222904 | 0.997718917 |
| **rs1402467_GC** | -0.07178529 | 0.356584494 | -0.01937853 | 0.803694779 | -0.04810668 | 0.53699493 |
| **rs1402467_GG** | 0.003974832 | 0.95934103 | -0.02396804 | 0.758498541 | 0.058802819 | 0.450341495 |
| **rs6599234_AA** | -0.16391971 | 0.03428207 | 0.049234253 | 0.527484039 | 0.047445179 | 0.542614119 |
| **rs6599234_TA** | -0.07360067 | 0.344521458 | 0.008407073 | 0.914131166 | -0.05443034 | 0.484783279 |
| **rs6599234_TT** | 0.154340324 | 0.046427585 | -0.0327662 | 0.674218673 | 0.030461115 | 0.695961017 |
| **rs12493607_CC** | 0.057249942 | 0.462413508 | 0.087366649 | 0.261567441 | 0.063234422 | 0.416881139 |
| **rs12493607_GC** | 0.043789079 | 0.574184869 | 0.057638733 | 0.459374381 | 0.002571393 | 0.973690388 |
| **rs12493607_GG** | -0.08460522 | 0.277001988 | -0.11960611 | 0.123664135 | -0.04605702 | 0.554499541 |
| **rs4533622_AA** | 0.056916149 | 0.465031562 | 0.053615722 | 0.491352697 | -0.11065179 | 0.154570574 |
| **rs4533622_AC** | 0.046983407 | 0.546553798 | 0.077855526 | 0.317273161 | 0.055528418 | 0.476003106 |
| **rs4533622_CC** | -0.08542806 | 0.272339875 | -0.09902308 | 0.202954048 | 0.084995067 | 0.274786506 |
| **rs16998073_AA** | 0.101257615 | 0.192896676 | -0.07170621 | 0.357115929 | 0.132926618 | 0.086805272 |
| **rs16998073_TA** | -0.0449102 | 0.564412782 | -0.00726879 | 0.925721495 | -0.15445564 | 0.046262424 |
| **rs16998073_TT** | -0.09802205 | 0.20758027 | 0.133335041 | 0.085832133 | 0.027553017 | 0.723746459 |
| **rs6050_AA** | 0.142775595 | 0.065672777 | 0.030806169 | 0.692690026 | -0.10455496 | 0.178726001 |
| **rs6050_AG** | -0.06454048 | 0.407304369 | -0.03026467 | 0.697825742 | -0.08759447 | 0.260320921 |
| **rs6050_GG** | 0.021751444 | 0.780235332 | 0.021609115 | 0.781637101 | 0.123690366 | 0.11126168 |
| **rs10520514_AA** | 0.035312211 | 0.650514045 | 0.011684376 | 0.88087001 | -0.09724421 | 0.211227007 |
| **rs10520514_TA** | -0.06180084 | 0.427542997 | 0.016978852 | 0.827598343 | -0.00257139 | 0.973690388 |
| **rs10520514_TT** | 0.03929865 | 0.614099126 | -0.03944843 | 0.61274837 | 0.13388648 | 0.084532115 |
| **rs1501908_CC** | -0.09656528 | 0.214447342 | 0.022658647 | 0.771317281 | 0.019369766 | 0.803781786 |
| **rs1501908_GC** | 0.058686194 | 0.451241943 | -0.04865415 | 0.532366489 | -0.03160793 | 0.685111574 |
| **rs1501908_GG** | -0.00280543 | 0.971296739 | 0.035164336 | 0.651881591 | 0.020184231 | 0.795708547 |
| **rs12654264_AA** | -0.09096287 | 0.24236509 | 0.072211184 | 0.353731079 | 0.009133491 | 0.906743858 |
| **rs12654264_TA** | 0.047058827 | 0.54590938 | -0.02181064 | 0.779652544 | 0.066661707 | 0.392030227 |
| **rs12654264_TT** | 0.021900472 | 0.778768347 | -0.03430731 | 0.659830057 | -0.07989646 | 0.304714049 |
| **rs1610696_CC** | 0.090021052 | 0.24729634 | -0.04999942 | 0.521078792 | -0.04508082 | 0.562932566 |
| **rs1610696_GC** | -0.06406735 | 0.410758516 | 0.171648664 | 0.026553649 | 0.024905076 | 0.749365039 |
| **rs1610696_GG** | 0.00464777 | 0.952464965 | -0.13687029 | 0.077769177 | 0.004757433 | 0.951344745 |
| **rs1063320_CC** | -0.03637841 | 0.64068859 | 0.168742297 | 0.029264217 | -0.01801526 | 0.817253728 |
| **rs1063320_GC** | 0.037893931 | 0.626829551 | -0.18329803 | 0.017736866 | -0.02636026 | 0.735250319 |
| **rs1063320_GG** | -0.00207464 | 0.978771615 | 0.022658647 | 0.771317281 | 0.077133175 | 0.321797665 |
| **rs6296_CC** | 0.063245991 | 0.416795735 | -0.0103425 | 0.894466823 | -0.21976939 | 0.004321491 |
| **rs6296_GC** | -0.10101995 | 0.193948801 | 0.121868234 | 0.116669035 | 0.085648936 | 0.271097557 |
| **rs6296_GG** | 0.048353596 | 0.534904982 | -0.10988886 | 0.15745119 | 0.08887646 | 0.253382433 |
| **rs1801132_CC** | 0.087687842 | 0.259811197 | 0.013369877 | 0.863840978 | -0.08656128 | 0.266006796 |
| **rs1801132_GC** | -0.06755479 | 0.385703848 | 0.087465637 | 0.261025323 | 0.104068853 | 0.180765454 |
| **rs1801132_GG** | -0.02742531 | 0.724975247 | -0.14840438 | 0.055618242 | -0.02807241 | 0.718756152 |
| **rs11755527_CC** | 0.04363413 | 0.575541673 | 0.040695497 | 0.601553297 | -0.14978017 | 0.053364327 |
| **rs11755527_GC** | -0.01770344 | 0.820362833 | 0.05530335 | 0.477795671 | 0.051076233 | 0.51213247 |
| **rs11755527_GG** | -0.03351207 | 0.667239668 | -0.13352395 | 0.085385001 | 0.128838699 | 0.097039088 |
| **rs713598_CC** | -0.00079725 | 0.991841513 | -0.14840438 | 0.055618242 | -0.17457528 | 0.024044267 |
| **rs713598_GC** | -0.07065604 | 0.364219708 | 0.067088435 | 0.38899966 | 0.032750735 | 0.674363722 |
| **rs713598_GG** | 0.069272642 | 0.373710132 | 0.035164336 | 0.651881591 | 0.086277693 | 0.267582135 |
| **rs6943555_AA** | -0.16187254 | 0.03662264 | 0.11463105 | 0.140185615 | -0.0124046 | 0.87358625 |
| **rs6943555_TA** | 0.067309301 | 0.38743666 | 0.121868234 | 0.116669035 | 0.01704763 | 0.826910911 |
| **rs6943555_TT** | 0.048757396 | 0.531495851 | -0.1978171 | 0.010391149 | -0.00771529 | 0.921173155 |
| **rs180242_AA** | -0.04866452 | 0.532279029 | -0.01169711 | 0.880741118 | 0.056655627 | 0.467080593 |
| **rs180242_TA** | -0.0332336 | 0.669841984 | 0.188784802 | 0.014552627 | -0.11932442 | 0.124557438 |
| **rs180242_TT** | 0.067309301 | 0.38743666 | -0.18806062 | 0.014942025 | 0.085648936 | 0.271097557 |
| **rs6968865_AA** | 0.037825772 | 0.627450095 | 0.032073376 | 0.68072637 | 0.004056738 | 0.958503938 |
| **rs6968865_TA** | -0.03484499 | 0.654838807 | -0.00751838 | 0.923178708 | -0.02636026 | 0.735250319 |
| **rs6968865_TT** | -0.00309044 | 0.968382205 | -0.03103711 | 0.690503983 | 0.028853658 | 0.711272285 |
| **rs4646244_AA** | 0.074017504 | 0.341788486 | -0.08665255 | 0.26550111 | 0.027837507 | 0.72101162 |
| **rs4646244_TA** | -0.16663626 | 0.031372754 | 0.049319733 | 0.526766519 | -0.01592709 | 0.83812658 |
| **rs4646244_TT** | 0.116066821 | 0.135253745 | 0.010866252 | 0.889155942 | -0.00340736 | 0.965141808 |
| **rs1537415_CC** | 0.128942068 | 0.096769022 | 0.11413509 | 0.141920719 | 0.178096155 | 0.021297796 |
| **rs1537415_GC** | -0.06406735 | 0.410758516 | -0.05565349 | 0.475008563 | -0.14280249 | 0.065621419 |
| **rs1537415_GG** | -0.12737724 | 0.100921596 | -0.11476481 | 0.139720432 | -0.07237842 | 0.352614533 |
| **rs7020673_CC** | -0.16000449 | 0.038874774 | 0.145166668 | 0.061233711 | 0.053080933 | 0.495691067 |
| **rs7020673_GC** | -0.04819724 | 0.536227966 | -0.06474475 | 0.405818353 | -0.00340736 | 0.965141808 |
| **rs7020673_GG** | 0.19623936 | 0.011031891 | -0.06467114 | 0.406353478 | -0.04482931 | 0.565115194 |
| **rs11014166_AA** | -0.03823551 | 0.623723643 | -0.00357963 | 0.963380588 | -0.04298418 | 0.581249261 |
| **rs11014166_TA** | -0.03115226 | 0.689414901 | -0.02596462 | 0.73907936 | 0.027545496 | 0.723818807 |
| **rs11014166_TT** | 0.116160709 | 0.134935928 | 0.04883048 | 0.530880003 | 0.027553017 | 0.723746459 |
| **rs12772424_AA** | 0.040469476 | 0.603575528 | -0.09896525 | 0.203219265 | 0.001800452 | 0.981576707 |
| **rs12772424_TA** | -0.00844667 | 0.91372828 | 0.005981563 | 0.938847152 | 0.060771185 | 0.435296939 |
| **rs12772424_TT** | -0.04866452 | 0.532279029 | 0.141512124 | 0.068122548 | -0.09594905 | 0.217400528 |
| **rs10995271_CC** | -0.01757737 | 0.821620702 | -0.06290666 | 0.41930497 | -0.03572807 | 0.646674498 |
| **rs10995271_GC** | -0.09649036 | 0.214804843 | -0.10988886 | 0.15745119 | 0.022467651 | 0.773192383 |
| **rs10995271_GG** | 0.11170769 | 0.150649467 | 0.15960323 | 0.039373444 | 0.004056738 | 0.958503938 |
| **rs187238_CC** | -0.00955276 | 0.902483737 | 0.039244546 | 0.614587353 | 0.102487247 | 0.187519502 |
| **rs187238_GC** | -0.09175623 | 0.238264688 | -0.06680621 | 0.391002433 | 0.078754524 | 0.311700228 |
| **rs187238_GG** | 0.095357386 | 0.220263109 | 0.048560524 | 0.533156607 | -0.1244337 | 0.10911287 |
| **rs1800955_CC** | -0.09096287 | 0.24236509 | 0.004652261 | 0.952419084 | 0.009133491 | 0.906743858 |
| **rs1800955_TC** | 0.068857369 | 0.376588331 | 0.007574942 | 0.922602529 | 0.028327036 | 0.716314003 |
| **rs1800955_TT** | 0.100653043 | 0.195581257 | -0.05887866 | 0.449756453 | -0.18074606 | 0.019413409 |
| **rs10848653_AA** | 0.142775595 | 0.065672777 | -0.08351895 | 0.283238765 | -0.10455496 | 0.178726001 |
| **rs10848653_AG** | -0.0397889 | 0.609682868 | 0.008970561 | 0.908400104 | 0.066269123 | 0.3948308 |
| **rs10848653_GG** | -0.1328061 | 0.087094111 | 0.102313743 | 0.188271522 | 0.033329061 | 0.668949496 |
| **rs7153648_CC** | -0.02656809 | 0.733241493 | -0.03474242 | 0.655789771 | -0.12181077 | 0.116842847 |
| **rs7153648_GC** | 0.161182796 | 0.037441031 | 0.013369877 | 0.863840978 | 0.14931316 | 0.054120779 |
| **rs7153648_GG** | -0.14389443 | 0.063563963 | 0.005515428 | 0.943604496 | -0.08116766 | 0.297059199 |
| **rs684513_CC** | 0.124435319 | 0.109108235 | -0.0120025 | 0.877651465 | 0.048224115 | 0.536000444 |
| **rs684513_GC** | -0.08319135 | 0.285137985 | -0.02808493 | 0.71863596 | 0.03273566 | 0.674505078 |
| **rs684513_GG** | -0.05664765 | 0.46714344 | 0.057696338 | 0.458925038 | -0.11594178 | 0.135677902 |
| **rs1558902_AA** | -0.04295226 | 0.581530214 | -0.02305282 | 0.767451726 | -0.06378292 | 0.412843317 |
| **rs1558902_TA** | -0.14395338 | 0.063454413 | 0.042704382 | 0.583714353 | 0.040339182 | 0.604742654 |
| **rs1558902_TT** | 0.169736322 | 0.028311751 | -0.02808493 | 0.71863596 | -0.0003968 | 0.995939408 |
| **rs3865188_AA** | -0.05000893 | 0.520999458 | 0.033752853 | 0.664992779 | 0.114899389 | 0.139253605 |
| **rs3865188_TA** | -0.00596289 | 0.939037709 | -0.0848869 | 0.275400025 | -0.01073675 | 0.890468618 |
| **rs3865188_TT** | 0.045874336 | 0.556072986 | 0.060573714 | 0.436793145 | -0.08024156 | 0.302623265 |
| **rs11869286_CC** | 0.00464777 | 0.952464965 | 0.062539423 | 0.422030479 | 0.070965046 | 0.362120432 |
| **rs11869286_GC** | 0.056078662 | 0.471636084 | -0.13307527 | 0.086450056 | -0.12640942 | 0.103559491 |
| **rs11869286_GG** | -0.09951363 | 0.200714357 | 0.114761965 | 0.13973032 | 0.089942794 | 0.247709199 |
| **rs4291_AA** | -0.0449102 | 0.564412782 | 0.096040829 | 0.216958845 | -0.01725302 | 0.824858816 |
| **rs4291_TA** | -0.09290303 | 0.232423696 | -0.16390156 | 0.034302241 | 0.020184231 | 0.795708547 |
| **rs4291_TT** | 0.171864677 | 0.026361062 | 0.11463105 | 0.140185615 | 0.034676499 | 0.656401294 |
| **rs519113_CC** | 0.067326312 | 0.387316437 | -0.00510536 | 0.947791334 | -0.10848448 | 0.162858835 |
| **rs519113_GC** | -0.03637841 | 0.64068859 | -0.03040939 | 0.696451848 | -0.15025915 | 0.052597586 |
| **rs519113_GG** | -0.00097844 | 0.989987366 | 0.033478166 | 0.667556337 | 0.211939389 | 0.005966875 |
| **rs11672691_AA** | -0.01340287 | 0.863508231 | -0.11194407 | 0.149782034 | -0.02834637 | 0.716128653 |
| **rs11672691_GG** | 0.013402871 | 0.863508231 | 0.111944074 | 0.149782034 | 0.028346373 | 0.716128653 |
| **rs1884613_CC** | 0.009637868 | 0.901619248 | 0.030954586 | 0.691284821 | 0.060771185 | 0.435296939 |
| **rs1884613_GC** | -0.00165454 | 0.983069539 | -0.00274171 | 0.97194842 | 0.043087443 | 0.580340683 |
| **rs1884613_GG** | -0.011185 | 0.885926189 | -0.03944843 | 0.61274837 | -0.1438656 | 0.063617609 |
| **rs5756506_CC** | 0.110095505 | 0.156667 | 0.039244546 | 0.614587353 | 0.102487247 | 0.187519502 |
| **rs5756506_GC** | -0.08235666 | 0.290015589 | 0.020113896 | 0.796404885 | -0.00863644 | 0.911797817 |
| **rs5756506_GG** | 0.015877456 | 0.838624144 | -0.02848791 | 0.714772502 | -0.00990419 | 0.898914986 |
| **rs713875_CC** | -0.00165454 | 0.983069539 | -0.10448973 | 0.178998679 | -0.05825908 | 0.454548271 |
| **rs713875_GC** | 0.043789079 | 0.574184869 | 0.132256318 | 0.088421489 | 0.002571393 | 0.973690388 |
| **rs713875_GG** | -0.06973088 | 0.370549876 | -0.0497466 | 0.523190746 | 0.089942794 | 0.247709199 |

**Supplementary Table 6:** Combination of genes and their effect on various biological pathways.

| **GeneSet** | **N** | **n** | **P-value** | **adjusted P** | **genes** |
| --- | --- | --- | --- | --- | --- |
| GOBP MEMBRANE DEPOLARIZATION DURING AV NODE CELL ACTION POTENTIAL | 6 | 3 | 5.46E-08 | 4.23E-04 | SCN5A, CACNB2, CACNA1C |
| GOBP AV NODE CELL TO BUNDLE OF HIS CELL SIGNALING | 11 | 3 | 4.48E-07 | 1.26E-03 | SCN5A, CACNB2, CACNA1C |
| GOBP AV NODE CELL TO BUNDLE OF HIS CELL COMMUNICATION | 14 | 3 | 9.85E-07 | 1.26E-03 | SCN5A, CACNB2, CACNA1C |
| GOBP CELLULAR RESPONSE TO ORGANIC CYCLIC COMPOUND | 558 | 8 | 1.00E-06 | 1.26E-03 | IL1B, CTNNB1, HTR1B, ESR1, DRD4, IL18, SIX1, ACE |
| GOBP MUSCLE CELL PROLIFERATION | 237 | 6 | 1.03E-06 | 1.26E-03 | TGFBR2, CTNNB1, HTR1B, TCF7L2, IL18, SIX1 |
| GOBP POSITIVE REGULATION OF EPITHELIAL TO MESENCHYMAL TRANSITION | 57 | 4 | 1.35E-06 | 1.26E-03 | IL1B, TGFBR2, CTNNB1, TCF7L2 |
| GOBP POSITIVE REGULATION OF MULTICELLULAR ORGANISMAL PROCESS | 1610 | 12 | 1.37E-06 | 1.26E-03 | PRDM16, IL1B, TGFBR2, CTNNB1, FGA, HLA-G, ZNF365, TCF7L2, IL18, SIX1, ACE, PVRL2 |
| GOBP EPITHELIAL CELL APOPTOTIC PROCESS | 138 | 5 | 1.55E-06 | 1.26E-03 | TGFBR2, FGA, HLA-G, ESR1, TCF7L2 |
| GOBP HOMEOSTATIC PROCESS | 1637 | 12 | 1.63E-06 | 1.26E-03 | PRDM16, IL1B, CTNNB1, HTR1B, CACNB2, TCF7L2, DRD4, IL18, CACNA1C, FTO, ACE, TMPRSS6 |
| GOBP CELL CELL SIGNALING | 1647 | 12 | 1.74E-06 | 1.26E-03 | IL1B, SCN5A, CTNNB1, FGA, HTR1B, CACNB2, TCF7L2, DRD4, IL18, CACNA1C, CHRNA5, ACE |
| GOBP RESPONSE TO XENOBIOTIC STIMULUS | 423 | 7 | 2.03E-06 | 1.26E-03 | SULT1C4, IL1B, TGFBR2, CTNNB1, HTR1B, NAT2, ACE |
| GOBP POSITIVE REGULATION OF CELL DEVELOPMENT | 426 | 7 | 2.13E-06 | 1.26E-03 | IL1B, TGFBR2, CTNNB1, HLA-G, ZNF365, IL18, ACE |
| GOBP POSITIVE REGULATION OF CELL DIFFERENTIATION | 848 | 9 | 2.29E-06 | 1.26E-03 | IL1B, TGFBR2, CTNNB1, HLA-G, ZNF365, TCF7L2, IL18, SIX1, ACE |
| GOBP POSITIVE REGULATION OF T CELL MEDIATED IMMUNITY | 66 | 4 | 2.44E-06 | 1.26E-03 | IL1B, HLA-G, IL18, PVRL2 |
| GOBP REGULATION OF CELL POPULATION PROLIFERATION | 1704 | 12 | 2.48E-06 | 1.26E-03 | IL1B, TGFBR2, SCN5A, CTNNB1, HLA-G, HTR1B, ESR1, TCF7L2, IL18, SIX1, FTO, ACE |
| GOBP ATRIAL CARDIAC MUSCLE CELL TO AV NODE CELL SIGNALING | 19 | 3 | 2.61E-06 | 1.26E-03 | SCN5A, CACNB2, CACNA1C |
| GOBP CELL PROLIFERATION INVOLVED IN HEART MORPHOGENESIS | 20 | 3 | 3.07E-06 | 1.40E-03 | TGFBR2, CTNNB1, SIX1 |
| GOBP RESPONSE TO OXYGEN CONTAINING COMPOUND | 1749 | 12 | 3.26E-06 | 1.40E-03 | IL1B, TGFBR2, SCN5A, CTNNB1, FGA, HTR1B, ESR1, TCF7L2, DRD4, IL18, SIX1, ACE |
| GOBP RESPONSE TO ORGANIC CYCLIC COMPOUND | 896 | 9 | 3.60E-06 | 1.47E-03 | IL1B, TGFBR2, CTNNB1, HTR1B, ESR1, DRD4, IL18, SIX1, ACE |
| GOBP CIRCULATORY SYSTEM DEVELOPMENT | 1166 | 10 | 3.80E-06 | 1.47E-03 | IL1B, TGFBR2, SCN5A, CTNNB1, HLA-G, TCF7L2, IL18, CACNA1C, SIX1, ACE |
| GOBP POSITIVE REGULATION OF TRANSCRIPTION BY RNA POLYMERASE II | 1184 | 10 | 4.36E-06 | 1.54E-03 | PRDM16, IL1B, CTNNB1, ESR1, AUTS2, GLIS3, TCF7L2, IL18, SIX1, TMPRSS6 |
| GOBP NEGATIVE REGULATION OF SECRETION | 171 | 5 | 4.45E-06 | 1.54E-03 | IL1B, HMGCR, HTR1B, DRD4, ACE |
| GOBP SMOOTH MUSCLE CELL PROLIFERATION | 172 | 5 | 4.58E-06 | 1.54E-03 | TGFBR2, CTNNB1, HTR1B, TCF7L2, IL18 |
| GOBP POSITIVE REGULATION OF MESENCHYMAL CELL PROLIFERATION | 24 | 3 | 5.42E-06 | 1.75E-03 | TGFBR2, CTNNB1, SIX1 |
| GOBP POSITIVE REGULATION OF CELL POPULATION PROLIFERATION | 950 | 9 | 5.79E-06 | 1.80E-03 | IL1B, TGFBR2, SCN5A, CTNNB1, HTR1B, ESR1, TCF7L2, IL18, SIX1 |
| GOBP MEMBRANE DEPOLARIZATION DURING ACTION POTENTIAL | 25 | 3 | 6.16E-06 | 1.84E-03 | SCN5A, CACNB2, CACNA1C |
| GOBP IMMUNE SYSTEM DEVELOPMENT | 196 | 5 | 8.65E-06 | 2.48E-03 | TGFBR2, CTNNB1, HLA-G, CACNA1C, SIX1 |
| GOBP LEUKOCYTE PROLIFERATION | 345 | 6 | 8.99E-06 | 2.49E-03 | IL1B, TGFBR2, CTNNB1, HLA-G, IL18, ACE |
| GOBP REGULATION OF T CELL MEDIATED IMMUNITY | 93 | 4 | 9.61E-06 | 2.57E-03 | IL1B, HLA-G, IL18, PVRL2 |
| GOBP REGULATION OF MESENCHYMAL CELL PROLIFERATION | 30 | 3 | 1.08E-05 | 2.79E-03 | TGFBR2, CTNNB1, SIX1 |
| GOBP CENTRAL NERVOUS SYSTEM DEVELOPMENT | 1040 | 9 | 1.20E-05 | 2.86E-03 | TMEM57, IL1B, TGFBR2, SCN5A, CTNNB1, PRDM8, ZNF365, SIX1, ACE |
| GOBP VASCULATURE DEVELOPMENT | 784 | 8 | 1.23E-05 | 2.86E-03 | IL1B, TGFBR2, CTNNB1, HLA-G, TCF7L2, IL18, SIX1, ACE |
| GOBP T CELL PROLIFERATION | 211 | 5 | 1.24E-05 | 2.86E-03 | IL1B, TGFBR2, CTNNB1, HLA-G, IL18 |
| GOBP RESPONSE TO SALT | 366 | 6 | 1.26E-05 | 2.86E-03 | IL1B, SCN5A, FGA, HTR1B, DRD4, SIX1 |
| GOBP REGULATION OF MONOATOMIC ION TRANSPORT | 576 | 7 | 1.53E-05 | 3.32E-03 | SCN5A, CTNNB1, HTR1B, CACNB2, DRD4, CACNA1C, ACE |
| GOBP NEGATIVE REGULATION OF SIGNALING | 1367 | 10 | 1.54E-05 | 3.32E-03 | PRDM16, IL1B, CTNNB1, FGA, HMGCR, HLA-G, HTR1B, ESR1, TCF7L2, TMPRSS6 |
| GOBP REGULATION OF EPITHELIAL TO MESENCHYMAL TRANSITION | 106 | 4 | 1.61E-05 | 3.38E-03 | IL1B, TGFBR2, CTNNB1, TCF7L2 |
| GOBP REGULATION OF EPITHELIAL CELL APOPTOTIC PROCESS | 108 | 4 | 1.74E-05 | 3.54E-03 | FGA, HLA-G, ESR1, TCF7L2 |
| GOBP DEVELOPMENTAL GROWTH INVOLVED IN MORPHOGENESIS | 229 | 5 | 1.83E-05 | 3.54E-03 | TGFBR2, CTNNB1, ESR1, AUTS2, SIX1 |
| GOBP REGULATION OF TRANSPORT | 1734 | 11 | 1.99E-05 | 3.54E-03 | IL1B, SCN5A, CTNNB1, FGA, HMGCR, HTR1B, CACNB2, TCF7L2, DRD4, CACNA1C, ACE |
| GOBP POSITIVE REGULATION OF PROTEOGLYCAN BIOSYNTHETIC PROCESS | 5 | 2 | 2.01E-05 | 3.54E-03 | CTNNB1, TCF7L2 |
| GOBP CRANIAL GANGLION DEVELOPMENT | 5 | 2 | 2.01E-05 | 3.54E-03 | CTNNB1, SIX1 |
| GOBP FUNGIFORM PAPILLA MORPHOGENESIS | 5 | 2 | 2.01E-05 | 3.54E-03 | CTNNB1, SIX1 |
| GOBP POSITIVE REGULATION OF SULFUR METABOLIC PROCESS | 5 | 2 | 2.01E-05 | 3.54E-03 | CTNNB1, TCF7L2 |
| GOBP CELL CELL SIGNALING INVOLVED IN CARDIAC CONDUCTION | 37 | 3 | 2.06E-05 | 3.54E-03 | SCN5A, CACNB2, CACNA1C |
| GOBP REGULATION OF SECRETION | 611 | 7 | 2.24E-05 | 3.74E-03 | IL1B, FGA, HMGCR, HTR1B, TCF7L2, DRD4, ACE |
| GOBP POSITIVE REGULATION OF MOLECULAR FUNCTION | 1429 | 10 | 2.27E-05 | 3.74E-03 | IL1B, TGFBR2, CTNNB1, ESR1, CACNB2, TCF7L2, DRD4, IL18, CACNA1C, ACE |
| GOBP REGULATION OF METAL ION TRANSPORT | 409 | 6 | 2.35E-05 | 3.80E-03 | SCN5A, CTNNB1, CACNB2, DRD4, CACNA1C, ACE |
| GOBP POSITIVE REGULATION OF RNA METABOLIC PROCESS | 1774 | 11 | 2.46E-05 | 3.87E-03 | PRDM16, IL1B, CTNNB1, ESR1, AUTS2, GLIS3, TCF7L2, IL18, SIX1, FTO, TMPRSS6 |
| GOBP T CELL MEDIATED IMMUNITY | 119 | 4 | 2.54E-05 | 3.87E-03 | IL1B, HLA-G, IL18, PVRL2 |
| GOBP RESPONSE TO ALCOHOL | 246 | 5 | 2.58E-05 | 3.87E-03 | TGFBR2, CTNNB1, HTR1B, DRD4, ACE |
| GOBP POSITIVE REGULATION OF LYMPHOCYTE MEDIATED IMMUNITY | 120 | 4 | 2.63E-05 | 3.87E-03 | IL1B, HLA-G, IL18, PVRL2 |
| GOBP MESENCHYMAL CELL DIFFERENTIATION | 248 | 5 | 2.69E-05 | 3.87E-03 | IL1B, TGFBR2, CTNNB1, TCF7L2, SIX1 |
| GOBP POSITIVE REGULATION OF ADAPTIVE IMMUNE RESPONSE | 122 | 4 | 2.81E-05 | 3.87E-03 | IL1B, HLA-G, IL18, PVRL2 |
| GOBP REGULATION OF HEART RATE BY CARDIAC CONDUCTION | 41 | 3 | 2.81E-05 | 3.87E-03 | SCN5A, CACNB2, CACNA1C |
| GOBP POSITIVE REGULATION OF DEFENSE RESPONSE | 423 | 6 | 2.84E-05 | 3.87E-03 | IL1B, HLA-G, ESR1, IL18, ACE, PVRL2 |
| GOBP REGULATION OF BLOOD CIRCULATION | 251 | 5 | 2.85E-05 | 3.87E-03 | SCN5A, FGA, CACNB2, CACNA1C, ACE |
| GOBP FUNGIFORM PAPILLA DEVELOPMENT | 6 | 2 | 3.01E-05 | 3.97E-03 | CTNNB1, SIX1 |
| GOBP MESENCHYMAL CELL PROLIFERATION | 42 | 3 | 3.02E-05 | 3.97E-03 | TGFBR2, CTNNB1, SIX1 |
| GOBP REGULATION OF CALCIUM ION TRANSPORT | 259 | 5 | 3.31E-05 | 4.27E-03 | CTNNB1, CACNB2, DRD4, CACNA1C, ACE |
| GOBP ADAPTIVE IMMUNE RESPONSE | 436 | 6 | 3.36E-05 | 4.27E-03 | IL1B, FGA, HLA-G, BACH2, IL18, PVRL2 |
| GOBP TUBE MORPHOGENESIS | 908 | 8 | 3.53E-05 | 4.31E-03 | IL1B, TGFBR2, CTNNB1, HLA-G, ESR1, IL18, SIX1, ACE |
| GOBP CALCIUM ION TRANSPORT | 440 | 6 | 3.54E-05 | 4.31E-03 | SCN5A, CTNNB1, CACNB2, DRD4, CACNA1C, ACE |
| GOBP REGULATION OF PROTEIN SECRETION | 263 | 5 | 3.56E-05 | 4.31E-03 | IL1B, FGA, HMGCR, TCF7L2, DRD4 |
| GOBP REGULATION OF LEUKOCYTE PROLIFERATION | 264 | 5 | 3.62E-05 | 4.32E-03 | IL1B, TGFBR2, CTNNB1, HLA-G, IL18 |
| GOBP ICOSANOID SECRETION | 46 | 3 | 3.98E-05 | 4.62E-03 | IL1B, DRD4, ACE |
| GOBP NEGATIVE REGULATION OF TRANSCRIPTION BY RNA POLYMERASE II | 924 | 8 | 4.00E-05 | 4.62E-03 | PRDM16, CTNNB1, BACH2, ESR1, GLIS3, TCF7L2, SIX1, TMPRSS6 |
| GOBP TRACHEA FORMATION | 7 | 2 | 4.21E-05 | 4.63E-03 | TGFBR2, CTNNB1 |
| GOBP ADENYLATE CYCLASE INHIBITING SEROTONIN RECEPTOR SIGNALING PATHWAY | 7 | 2 | 4.21E-05 | 4.63E-03 | HTR1B, DRD4 |
| GOBP CALCIUM ION IMPORT | 47 | 3 | 4.25E-05 | 4.63E-03 | CTNNB1, CACNB2, ACE |
| GOBP REGULATION OF CELL DIFFERENTIATION | 1538 | 10 | 4.26E-05 | 4.63E-03 | IL1B, TGFBR2, CTNNB1, HLA-G, ZNF365, TCF7L2, IL18, SIX1, FTO, ACE |
| GOBP BLOOD VESSEL MORPHOGENESIS | 677 | 7 | 4.30E-05 | 4.63E-03 | IL1B, TGFBR2, CTNNB1, HLA-G, IL18, SIX1, ACE |
| GOBP POSITIVE REGULATION OF LEUKOCYTE MEDIATED IMMUNITY | 142 | 4 | 5.08E-05 | 5.39E-03 | IL1B, HLA-G, IL18, PVRL2 |
| GOBP REGULATION OF MORPHOGENESIS OF A BRANCHING STRUCTURE | 51 | 3 | 5.43E-05 | 5.69E-03 | CTNNB1, ESR1, SIX1 |
| GOBP CELLULAR RESPONSE TO OXYGEN CONTAINING COMPOUND | 1262 | 9 | 5.52E-05 | 5.71E-03 | IL1B, SCN5A, CTNNB1, HTR1B, ESR1, DRD4, IL18, SIX1, ACE |
| GOBP T HELPER 1 CELL CYTOKINE PRODUCTION | 8 | 2 | 5.61E-05 | 5.72E-03 | IL1B, IL18 |
| GOBP T CELL DIFFERENTIATION | 292 | 5 | 5.85E-05 | 5.88E-03 | IL1B, TGFBR2, CTNNB1, HLA-G, IL18 |
| GOBP ADAPTIVE IMMUNE RESPONSE BASED ON SOMATIC RECOMBINATION OF IMMUNE RECEPTORS BUILT FROM IMMUNOGLOBULIN SUPERFAMILY DOMAINS | 293 | 5 | 5.94E-05 | 5.90E-03 | IL1B, HLA-G, BACH2, IL18, PVRL2 |
| GOBP CARDIAC MUSCLE CELL ACTION POTENTIAL INVOLVED IN CONTRACTION | 53 | 3 | 6.10E-05 | 5.91E-03 | SCN5A, CACNB2, CACNA1C |
| **GOBP BROWN FAT CELL DIFFERENTIATION** | **53** | **3** | **6.10E-05** | **5.91E-03** | **PRDM16, SIX1, FTO** |
| GOBP POSITIVE REGULATION OF TRANSFERASE ACTIVITY | 491 | 6 | 6.51E-05 | 6.19E-03 | IL1B, TGFBR2, CTNNB1, DRD4, IL18, ACE |
| GOBP CHEMICAL HOMEOSTASIS | 991 | 8 | 6.55E-05 | 6.19E-03 | HTR1B, CACNB2, TCF7L2, DRD4, IL18, CACNA1C, ACE, TMPRSS6 |
| GOBP BRANCHING MORPHOGENESIS OF AN EPITHELIAL TUBE | 153 | 4 | 6.79E-05 | 6.34E-03 | TGFBR2, CTNNB1, ESR1, SIX1 |
| GOBP MESENCHYME DEVELOPMENT | 303 | 5 | 6.96E-05 | 6.42E-03 | IL1B, TGFBR2, CTNNB1, TCF7L2, SIX1 |
| GOBP POSITIVE REGULATION OF DEVELOPMENTAL PROCESS | 1304 | 9 | 7.12E-05 | 6.42E-03 | IL1B, TGFBR2, CTNNB1, HLA-G, ZNF365, TCF7L2, IL18, SIX1, ACE |
| GOBP POSITIVE REGULATION OF T CELL TOLERANCE INDUCTION | 9 | 2 | 7.21E-05 | 6.42E-03 | TGFBR2, HLA-G |
| GOBP REGULATION OF SECONDARY HEART FIELD CARDIOBLAST PROLIFERATION | 9 | 2 | 7.21E-05 | 6.42E-03 | CTNNB1, SIX1 |
| GOBP NEGATIVE REGULATION OF RESPONSE TO STIMULUS | 1640 | 10 | 7.34E-05 | 6.47E-03 | PRDM16, IL1B, CTNNB1, FGA, HMGCR, HLA-G, HTR1B, ESR1, TCF7L2, TMPRSS6 |
| GOBP POSITIVE REGULATION OF CELL CELL ADHESION | 312 | 5 | 7.99E-05 | 6.96E-03 | IL1B, TGFBR2, FGA, HLA-G, IL18 |
| GOBP CELL COMMUNICATION INVOLVED IN CARDIAC CONDUCTION | 59 | 3 | 8.42E-05 | 7.25E-03 | SCN5A, CACNB2, CACNA1C |
| GOBP POSITIVE REGULATION OF CATALYTIC ACTIVITY | 1035 | 8 | 8.88E-05 | 7.36E-03 | IL1B, TGFBR2, CTNNB1, ESR1, DRD4, IL18, CACNA1C, ACE |
| GOBP REGULATION OF GAP JUNCTION ASSEMBLY | 10 | 2 | 9.00E-05 | 7.36E-03 | IL1B, ACE |
| GOBP CARDIOBLAST PROLIFERATION | 10 | 2 | 9.00E-05 | 7.36E-03 | CTNNB1, SIX1 |
| GOBP TONGUE MORPHOGENESIS | 10 | 2 | 9.00E-05 | 7.36E-03 | CTNNB1, SIX1 |
| GOBP SYNAPTIC SIGNALING | 762 | 7 | 9.05E-05 | 7.36E-03 | IL1B, CTNNB1, HTR1B, CACNB2, DRD4, CHRNA5, ACE |
| GOBP REGULATION OF HORMONE LEVELS | 522 | 6 | 9.12E-05 | 7.36E-03 | IL1B, FGA, ESR1, TCF7L2, STARD3, ACE |
| GOBP EPITHELIAL TO MESENCHYMAL TRANSITION | 167 | 4 | 9.54E-05 | 7.49E-03 | IL1B, TGFBR2, CTNNB1, TCF7L2 |
| GOBP REGULATION OF INTRACELLULAR SIGNAL TRANSDUCTION | 1692 | 10 | 9.54E-05 | 7.49E-03 | IL1B, CTNNB1, FGA, HMGCR, HLA-G, ESR1, AUTS2, TCF7L2, DRD4, IL18 |
| GOBP INORGANIC ION HOMEOSTASIS | 527 | 6 | 9.61E-05 | 7.49E-03 | HTR1B, CACNB2, DRD4, CACNA1C, ACE, TMPRSS6 |
| GOBP REGULATION OF RESPONSE TO EXTERNAL STIMULUS | 1048 | 8 | 9.69E-05 | 7.49E-03 | IL1B, TGFBR2, FGA, HLA-G, ESR1, IL18, ACE, PVRL2 |
| GOBP ICOSANOID TRANSPORT | 62 | 3 | 9.76E-05 | 7.49E-03 | IL1B, DRD4, ACE |
| GOBP RESPONSE TO ENDOGENOUS STIMULUS | 1703 | 10 | 1.01E-04 | 7.65E-03 | PRDM16, IL1B, TGFBR2, CTNNB1, HTR1B, ESR1, DRD4, SIX1, ACE, TMPRSS6 |
| GOBP TISSUE REMODELING | 172 | 4 | 1.07E-04 | 8.04E-03 | CTNNB1, HTR1B, IL18, ACE |
| GOBP TRACHEA MORPHOGENESIS | 11 | 2 | 1.10E-04 | 8.19E-03 | TGFBR2, CTNNB1 |
| GOBP REGULATION OF MORPHOGENESIS OF AN EPITHELIUM | 65 | 3 | 1.12E-04 | 8.30E-03 | CTNNB1, ESR1, SIX1 |
| GOBP ANIMAL ORGAN FORMATION | 66 | 3 | 1.18E-04 | 8.60E-03 | TGFBR2, CTNNB1, SIX1 |
| GOBP REGULATION OF LYMPHOCYTE MEDIATED IMMUNITY | 177 | 4 | 1.19E-04 | 8.65E-03 | IL1B, HLA-G, IL18, PVRL2 |
| GOBP TEMPERATURE HOMEOSTASIS | 178 | 4 | 1.22E-04 | 8.75E-03 | PRDM16, IL1B, IL18, FTO |
| GOBP REGULATION OF CELL DEVELOPMENT | 802 | 7 | 1.25E-04 | 8.85E-03 | IL1B, TGFBR2, CTNNB1, HLA-G, ZNF365, IL18, ACE |
| GOBP REGULATION OF TRANSFERASE ACTIVITY | 809 | 7 | 1.31E-04 | 9.19E-03 | IL1B, TGFBR2, CTNNB1, HMGCR, DRD4, IL18, ACE |
| GOBP POSITIVE REGULATION OF TOLERANCE INDUCTION | 12 | 2 | 1.32E-04 | 9.19E-03 | TGFBR2, HLA-G |
| GOBP CELLULAR RESPONSE TO XENOBIOTIC STIMULUS | 182 | 4 | 1.33E-04 | 9.19E-03 | SULT1C4, IL1B, HTR1B, NAT2 |
| GOBP POSITIVE REGULATION OF RESPONSE TO EXTERNAL STIMULUS | 560 | 6 | 1.34E-04 | 9.19E-03 | IL1B, HLA-G, ESR1, IL18, ACE, PVRL2 |
| GOBP CRANIAL SKELETAL SYSTEM DEVELOPMENT | 71 | 3 | 1.46E-04 | 9.94E-03 | TGFBR2, CTNNB1, SIX1 |
| GOBP TUBE DEVELOPMENT | 1114 | 8 | 1.48E-04 | 9.97E-03 | IL1B, TGFBR2, CTNNB1, HLA-G, ESR1, IL18, SIX1, ACE |
| GOBP NEGATIVE REGULATION OF PROTEIN SECRETION | 72 | 3 | 1.52E-04 | 1.01E-02 | IL1B, HMGCR, DRD4 |
| GOBP REGULATION OF SYSTEM PROCESS | 574 | 6 | 1.53E-04 | 1.01E-02 | SCN5A, FGA, CACNB2, CACNA1C, FTO, ACE |
| GOBP ECTODERMAL PLACODE DEVELOPMENT | 13 | 2 | 1.56E-04 | 1.02E-02 | CTNNB1, SIX1 |
| GOBP CIRCULATORY SYSTEM PROCESS | 580 | 6 | 1.62E-04 | 1.05E-02 | SCN5A, FGA, HTR1B, CACNB2, CACNA1C, ACE |
| GOBP REGULATION OF T CELL ACTIVATION | 364 | 5 | 1.64E-04 | 1.05E-02 | IL1B, TGFBR2, CTNNB1, HLA-G, IL18 |
| GOBP MEMBRANE DEPOLARIZATION | 74 | 3 | 1.65E-04 | 1.05E-02 | SCN5A, CACNB2, CACNA1C |
| GOBP REGULATION OF RESPONSE TO STRESS | 1456 | 9 | 1.65E-04 | 1.05E-02 | IL1B, CTNNB1, FGA, HLA-G, ESR1, ZNF365, IL18, ACE, PVRL2 |
| GOBP HEART DEVELOPMENT | 584 | 6 | 1.68E-04 | 1.06E-02 | TGFBR2, SCN5A, CTNNB1, CACNA1C, SIX1, ACE |
| GOBP PROTEIN LOCALIZATION TO EXTRACELLULAR REGION | 367 | 5 | 1.71E-04 | 1.07E-02 | IL1B, FGA, HMGCR, TCF7L2, DRD4 |
| GOBP CARDIAC MUSCLE CELL CONTRACTION | 75 | 3 | 1.72E-04 | 1.07E-02 | SCN5A, CACNB2, CACNA1C |
| GOBP REGULATION OF ADAPTIVE IMMUNE RESPONSE | 196 | 4 | 1.77E-04 | 1.08E-02 | IL1B, HLA-G, IL18, PVRL2 |
| GOBP POSITIVE REGULATION OF MACROMOLECULE BIOSYNTHETIC PROCESS | 1823 | 10 | 1.77E-04 | 1.08E-02 | PRDM16, IL1B, CTNNB1, ESR1, AUTS2, GLIS3, TCF7L2, IL18, SIX1, TMPRSS6 |
| GOBP POSITIVE REGULATION OF MYOBLAST PROLIFERATION | 14 | 2 | 1.81E-04 | 1.08E-02 | CTNNB1, SIX1 |
| GOBP REGULATION OF SULFUR METABOLIC PROCESS | 14 | 2 | 1.81E-04 | 1.08E-02 | CTNNB1, TCF7L2 |
| GOBP POSITIVE REGULATION OF GRANULOCYTE MACROPHAGE COLONY STIMULATING FACTOR PRODUCTION | 14 | 2 | 1.81E-04 | 1.08E-02 | IL1B, IL18 |
| GOBP MONOATOMIC ION HOMEOSTASIS | 593 | 6 | 1.83E-04 | 1.08E-02 | HTR1B, CACNB2, DRD4, CACNA1C, ACE, TMPRSS6 |
| GOBP REGULATION OF TRANSMEMBRANE TRANSPORT | 594 | 6 | 1.84E-04 | 1.08E-02 | IL1B, SCN5A, CACNB2, DRD4, CACNA1C, ACE |
| GOBP CARDIAC MUSCLE CELL ACTION POTENTIAL | 77 | 3 | 1.86E-04 | 1.08E-02 | SCN5A, CACNB2, CACNA1C |
| GOBP MORPHOGENESIS OF A BRANCHING STRUCTURE | 199 | 4 | 1.87E-04 | 1.08E-02 | TGFBR2, CTNNB1, ESR1, SIX1 |
| GOBP VASOCONSTRICTION | 78 | 3 | 1.93E-04 | 1.10E-02 | FGA, HTR1B, ACE |
| GOBP POSITIVE REGULATION OF CYTOKINE PRODUCTION INVOLVED IN IMMUNE RESPONSE | 78 | 3 | 1.93E-04 | 1.10E-02 | IL1B, HLA-G, IL18 |
| GOBP RESPIRATORY SYSTEM DEVELOPMENT | 202 | 4 | 1.98E-04 | 1.12E-02 | TGFBR2, CTNNB1, SIX1, ACE |
| GOBP NATURAL KILLER CELL MEDIATED IMMUNITY | 79 | 3 | 2.01E-04 | 1.13E-02 | HLA-G, IL18, PVRL2 |
| GOBP LEUKOCYTE MEDIATED IMMUNITY | 382 | 5 | 2.06E-04 | 1.14E-02 | IL1B, HLA-G, IL18, ACE, PVRL2 |
| GOBP POSITIVE REGULATION OF CELL ACTIVATION | 382 | 5 | 2.06E-04 | 1.14E-02 | IL1B, TGFBR2, HLA-G, IL18, PVRL2 |
| GOBP T CELL TOLERANCE INDUCTION | 15 | 2 | 2.09E-04 | 1.14E-02 | TGFBR2, HLA-G |
| GOBP POSITIVE REGULATION OF HETEROTYPIC CELL CELL ADHESION | 15 | 2 | 2.09E-04 | 1.14E-02 | IL1B, FGA |
| GOBP PROTEIN KINASE B SIGNALING | 205 | 4 | 2.10E-04 | 1.14E-02 | IL1B, HLA-G, TCF7L2, IL18 |
| GOBP OUTFLOW TRACT MORPHOGENESIS | 81 | 3 | 2.16E-04 | 1.16E-02 | TGFBR2, CTNNB1, SIX1 |
| GOBP MULTI MULTICELLULAR ORGANISM PROCESS | 209 | 4 | 2.26E-04 | 1.20E-02 | IL1B, TGFBR2, ESR1, ACE |
| GOBP REGULATION OF CELL JUNCTION ASSEMBLY | 209 | 4 | 2.26E-04 | 1.20E-02 | IL1B, CTNNB1, SIX1, ACE |
| **GOBP RESPONSE TO LIPID** | **885** | **7** | **2.28E-04** | **1.20E-02** | **IL1B, TGFBR2, CTNNB1, HTR1B, ESR1, IL18, ACE** |
| GOBP MONOATOMIC ION TRANSPORT | 1200 | 8 | 2.46E-04 | 1.29E-02 | SCN5A, CTNNB1, HTR1B, CACNB2, DRD4, CACNA1C, CHRNA5, ACE |
| GOBP ANATOMICAL STRUCTURE FORMATION INVOLVED IN MORPHOGENESIS | 1206 | 8 | 2.55E-04 | 1.32E-02 | IL1B, TGFBR2, CTNNB1, HLA-G, IL18, SIX1, ACE, PVRL2 |
| GOBP DEVELOPMENTAL GROWTH | 631 | 6 | 2.56E-04 | 1.32E-02 | TGFBR2, CTNNB1, ESR1, AUTS2, SIX1, FTO |
| GOBP REGULATION OF CELL ACTIVATION | 635 | 6 | 2.64E-04 | 1.34E-02 | IL1B, TGFBR2, CTNNB1, HLA-G, IL18, PVRL2 |
| GOBP POSITIVE REGULATION OF KINASE ACTIVITY | 404 | 5 | 2.67E-04 | 1.34E-02 | IL1B, TGFBR2, DRD4, IL18, ACE |
| GOBP REGULATION OF PRESYNAPTIC CYTOSOLIC CALCIUM ION CONCENTRATION | 17 | 2 | 2.70E-04 | 1.34E-02 | HTR1B, CACNB2 |
| GOBP GANGLION DEVELOPMENT | 17 | 2 | 2.70E-04 | 1.34E-02 | CTNNB1, SIX1 |
| GOBP BRANCH ELONGATION OF AN EPITHELIUM | 17 | 2 | 2.70E-04 | 1.34E-02 | ESR1, SIX1 |
| GOBP GRANULOCYTE MACROPHAGE COLONY STIMULATING FACTOR PRODUCTION | 17 | 2 | 2.70E-04 | 1.34E-02 | IL1B, IL18 |
| GOBP REGULATION OF STEM CELL PROLIFERATION | 88 | 3 | 2.76E-04 | 1.36E-02 | TGFBR2, CTNNB1, ACE |
| GOBP NEGATIVE REGULATION OF APOPTOTIC SIGNALING PATHWAY | 222 | 4 | 2.84E-04 | 1.39E-02 | IL1B, CTNNB1, FGA, TCF7L2 |
| GOBP CELL CELL ADHESION | 922 | 7 | 2.93E-04 | 1.41E-02 | IL1B, TGFBR2, CTNNB1, FGA, HLA-G, IL18, PVRL2 |
| GOBP POSITIVE REGULATION OF PROTEIN MODIFICATION PROCESS | 923 | 7 | 2.95E-04 | 1.41E-02 | IL1B, TGFBR2, CTNNB1, AUTS2, DRD4, IL18, ACE |
| GOBP SECRETION | 927 | 7 | 3.03E-04 | 1.41E-02 | IL1B, FGA, HMGCR, HTR1B, TCF7L2, DRD4, ACE |
| GOBP POSITIVE REGULATION OF NEUROINFLAMMATORY RESPONSE | 18 | 2 | 3.04E-04 | 1.41E-02 | IL1B, IL18 |
| GOBP TRACHEA DEVELOPMENT | 18 | 2 | 3.04E-04 | 1.41E-02 | TGFBR2, CTNNB1 |
| GOBP POSITIVE REGULATION OF HISTONE H3 K4 METHYLATION | 18 | 2 | 3.04E-04 | 1.41E-02 | CTNNB1, AUTS2 |
| GOBP POSITIVE REGULATION OF T HELPER 1 TYPE IMMUNE RESPONSE | 18 | 2 | 3.04E-04 | 1.41E-02 | IL1B, IL18 |
| GOBP GAP JUNCTION ASSEMBLY | 18 | 2 | 3.04E-04 | 1.41E-02 | IL1B, ACE |
| GOBP EMBRYONIC SKELETAL SYSTEM MORPHOGENESIS | 91 | 3 | 3.05E-04 | 1.41E-02 | TGFBR2, CTNNB1, SIX1 |
| GOBP REGULATION OF CELLULAR LOCALIZATION | 930 | 7 | 3.09E-04 | 1.42E-02 | IL1B, CTNNB1, FGA, HMGCR, TCF7L2, DRD4, SIX1 |
| GOBP POSITIVE REGULATION OF NEUROGENESIS | 227 | 4 | 3.09E-04 | 1.42E-02 | IL1B, CTNNB1, ZNF365, ACE |
| GOBP NEGATIVE REGULATION OF RNA BIOSYNTHETIC PROCESS | 1244 | 8 | 3.14E-04 | 1.43E-02 | PRDM16, CTNNB1, BACH2, ESR1, GLIS3, TCF7L2, SIX1, TMPRSS6 |
| GOBP RESPONSE TO CARBOHYDRATE | 229 | 4 | 3.20E-04 | 1.45E-02 | IL1B, TGFBR2, TCF7L2, ACE |
| GOBP REGULATION OF MEMBRANE POTENTIAL | 423 | 5 | 3.29E-04 | 1.47E-02 | SCN5A, CACNB2, DRD4, CACNA1C, CHRNA5 |
| GOBP HORMONE METABOLIC PROCESS | 231 | 4 | 3.30E-04 | 1.47E-02 | ESR1, TCF7L2, STARD3, ACE |
| GOBP POSITIVE REGULATION OF SMOOTH MUSCLE CELL PROLIFERATION | 94 | 3 | 3.35E-04 | 1.47E-02 | TGFBR2, HTR1B, IL18 |
| GOBP GLIAL CELL DIFFERENTIATION | 232 | 4 | 3.36E-04 | 1.47E-02 | IL1B, CTNNB1, PRDM8, ZNF365 |
| GOBP TONGUE DEVELOPMENT | 19 | 2 | 3.39E-04 | 1.47E-02 | CTNNB1, SIX1 |
| GOBP CD4 POSITIVE ALPHA BETA T CELL CYTOKINE PRODUCTION | 19 | 2 | 3.39E-04 | 1.47E-02 | IL1B, IL18 |
| **GOBP CALCIUM ION TRANSMEMBRANE TRANSPORT VIA HIGH VOLTAGE GATED CALCIUM CHANNEL** | **19** | **2** | **3.39E-04** | **1.47E-02** | **CACNB2, CACNA1C** |
| **GOBP NEGATIVE REGULATION OF GLUCOSE TRANSMEMBRANE TRANSPORT** | **19** | **2** | **3.39E-04** | **1.47E-02** | **IL1B, ACE** |
| GOBP CARDIAC CONDUCTION | 96 | 3 | 3.57E-04 | 1.53E-02 | SCN5A, CACNB2, CACNA1C |
| GOBP NEGATIVE REGULATION OF EXTRINSIC APOPTOTIC SIGNALING PATHWAY | 96 | 3 | 3.57E-04 | 1.53E-02 | IL1B, FGA, TCF7L2 |
| GOBP REGULATION OF LEUKOCYTE MEDIATED IMMUNITY | 238 | 4 | 3.70E-04 | 1.57E-02 | IL1B, HLA-G, IL18, PVRL2 |
| GOBP FAT CELL DIFFERENTIATION | 238 | 4 | 3.70E-04 | 1.57E-02 | PRDM16, TCF7L2, SIX1, FTO |
| GOBP NEPHRON TUBULE FORMATION | 20 | 2 | 3.77E-04 | 1.58E-02 | CTNNB1, SIX1 |
| GOBP REGULATION OF TOLERANCE INDUCTION | 20 | 2 | 3.77E-04 | 1.58E-02 | TGFBR2, HLA-G |
| GOBP NEGATIVE REGULATION OF BIOSYNTHETIC PROCESS | 1628 | 9 | 3.81E-04 | 1.59E-02 | PRDM16, CTNNB1, BACH2, ESR1, GLIS3, TCF7L2, SIX1, FTO, TMPRSS6 |
| GOBP REGULATION OF KINASE ACTIVITY | 682 | 6 | 3.87E-04 | 1.60E-02 | IL1B, TGFBR2, HMGCR, DRD4, IL18, ACE |
| GOBP OLIGODENDROCYTE DIFFERENTIATION | 99 | 3 | 3.90E-04 | 1.61E-02 | CTNNB1, PRDM8, ZNF365 |
| GOBP STEM CELL DIFFERENTIATION | 244 | 4 | 4.06E-04 | 1.67E-02 | CTNNB1, ESR1, SIX1, ACE |
| GOBP POSITIVE REGULATION OF INTRACELLULAR SIGNAL TRANSDUCTION | 977 | 7 | 4.16E-04 | 1.70E-02 | IL1B, CTNNB1, FGA, AUTS2, TCF7L2, DRD4, IL18 |
| GOBP REGULATION OF DNA BINDING TRANSCRIPTION FACTOR ACTIVITY | 447 | 5 | 4.24E-04 | 1.71E-02 | IL1B, CTNNB1, ESR1, TCF7L2, IL18 |
| GOBP HEART PROCESS | 247 | 4 | 4.26E-04 | 1.71E-02 | SCN5A, CACNB2, CACNA1C, ACE |
| GOBP REGULATION OF HEART RATE | 102 | 3 | 4.26E-04 | 1.71E-02 | SCN5A, CACNB2, CACNA1C |
| GOBP ACTIN MEDIATED CELL CONTRACTION | 103 | 3 | 4.38E-04 | 1.75E-02 | SCN5A, CACNB2, CACNA1C |
| GOBP POSITIVE REGULATION OF T CELL PROLIFERATION | 104 | 3 | 4.51E-04 | 1.79E-02 | IL1B, TGFBR2, IL18 |
| GOBP NEGATIVE REGULATION OF TRANSPORT | 460 | 5 | 4.83E-04 | 1.91E-02 | IL1B, HMGCR, HTR1B, DRD4, ACE |
| GOBP REGULATION OF GLIOGENESIS | 107 | 3 | 4.90E-04 | 1.93E-02 | IL1B, CTNNB1, ZNF365 |
| GOBP MONONUCLEAR CELL DIFFERENTIATION | 463 | 5 | 4.98E-04 | 1.94E-02 | IL1B, TGFBR2, CTNNB1, HLA-G, IL18 |
| GOBP CELL JUNCTION ORGANIZATION | 716 | 6 | 5.01E-04 | 1.94E-02 | IL1B, CTNNB1, CACNB2, ZNF365, SIX1, ACE |
| GOBP POSITIVE REGULATION OF IMMUNE EFFECTOR PROCESS | 258 | 4 | 5.02E-04 | 1.94E-02 | IL1B, HLA-G, IL18, PVRL2 |
| GOBP POSITIVE REGULATION OF DNA BINDING TRANSCRIPTION FACTOR ACTIVITY | 261 | 4 | 5.24E-04 | 2.02E-02 | IL1B, CTNNB1, ESR1, IL18 |
| GOBP POSITIVE REGULATION OF CELL ADHESION | 470 | 5 | 5.33E-04 | 2.04E-02 | IL1B, TGFBR2, FGA, HLA-G, IL18 |
| GOBP REGULATION OF MYOBLAST PROLIFERATION | 24 | 2 | 5.45E-04 | 2.05E-02 | CTNNB1, SIX1 |
| **GOBP REGULATION OF BROWN FAT CELL DIFFERENTIATION** | **24** | **2** | **5.45E-04** | **2.05E-02** | **SIX1, FTO** |
| GOBP REGULATION OF HISTONE H3 K4 METHYLATION | 24 | 2 | 5.45E-04 | 2.05E-02 | CTNNB1, AUTS2 |
| GOBP ORGAN INDUCTION | 24 | 2 | 5.45E-04 | 2.05E-02 | CTNNB1, SIX1 |
| GOBP POSITIVE REGULATION OF LEUKOCYTE CELL CELL ADHESION | 264 | 4 | 5.47E-04 | 2.05E-02 | IL1B, TGFBR2, HLA-G, IL18 |
| GOBP POSITIVE REGULATION OF CELL COMMUNICATION | 1713 | 9 | 5.54E-04 | 2.06E-02 | IL1B, TGFBR2, CTNNB1, FGA, AUTS2, TCF7L2, DRD4, IL18, PVRL2 |
| GOBP EPITHELIAL CELL PROLIFERATION | 476 | 5 | 5.64E-04 | 2.09E-02 | SCN5A, CTNNB1, ESR1, TCF7L2, SIX1 |
| GOBP FATTY ACID TRANSPORT | 114 | 3 | 5.90E-04 | 2.16E-02 | IL1B, DRD4, ACE |
| GOBP REGULATION OF CELL CELL ADHESION | 481 | 5 | 5.91E-04 | 2.16E-02 | IL1B, TGFBR2, FGA, HLA-G, IL18 |
| GOBP POSITIVE REGULATION OF ALPHA BETA T CELL PROLIFERATION | 25 | 2 | 5.92E-04 | 2.16E-02 | TGFBR2, IL18 |
| GOBP REGULATION OF DEFENSE RESPONSE | 740 | 6 | 5.96E-04 | 2.16E-02 | IL1B, HLA-G, ESR1, IL18, ACE, PVRL2 |
| **GOBP LIPID LOCALIZATION** | **482** | **5** | **5.97E-04** | **2.16E-02** | **IL1B, DRD4, FTO, STARD3, ACE** |
| GOBP STEM CELL PROLIFERATION | 116 | 3 | 6.20E-04 | 2.24E-02 | TGFBR2, CTNNB1, ACE |
| GOBP REGULATION OF LYMPHOCYTE ACTIVATION | 487 | 5 | 6.26E-04 | 2.24E-02 | IL1B, TGFBR2, CTNNB1, HLA-G, IL18 |
| GOBP POSITIVE REGULATION OF NERVOUS SYSTEM DEVELOPMENT | 274 | 4 | 6.29E-04 | 2.25E-02 | IL1B, CTNNB1, ZNF365, ACE |
| GOBP REGULATION OF CATALYTIC ACTIVITY | 1747 | 9 | 6.39E-04 | 2.25E-02 | IL1B, TGFBR2, CTNNB1, HMGCR, ESR1, DRD4, IL18, CACNA1C, ACE |
| GOBP POSITIVE REGULATION OF T CELL CYTOKINE PRODUCTION | 26 | 2 | 6.41E-04 | 2.25E-02 | IL1B, IL18 |
| GOBP POSITIVE REGULATION OF GLYCOPROTEIN METABOLIC PROCESS | 26 | 2 | 6.41E-04 | 2.25E-02 | CTNNB1, TCF7L2 |
| GOBP REGULATION OF HETEROTYPIC CELL CELL ADHESION | 26 | 2 | 6.41E-04 | 2.25E-02 | IL1B, FGA |
| GOBP CYTOKINE PRODUCTION INVOLVED IN IMMUNE RESPONSE | 118 | 3 | 6.52E-04 | 2.28E-02 | IL1B, HLA-G, IL18 |
| GOBP POSITIVE REGULATION OF PROTEIN METABOLIC PROCESS | 1391 | 8 | 6.63E-04 | 2.30E-02 | IL1B, TGFBR2, CTNNB1, AUTS2, TCF7L2, DRD4, IL18, ACE |
| GOBP CELL ACTIVATION | 1059 | 7 | 6.74E-04 | 2.33E-02 | IL1B, TGFBR2, CTNNB1, FGA, HLA-G, IL18, PVRL2 |
| GOBP INTRACELLULAR MONOATOMIC ION HOMEOSTASIS | 497 | 5 | 6.86E-04 | 2.36E-02 | HTR1B, CACNB2, DRD4, CACNA1C, TMPRSS6 |
| GOBP AXIS ELONGATION | 27 | 2 | 6.91E-04 | 2.37E-02 | ESR1, SIX1 |
| GOBP LYMPHOCYTE MEDIATED IMMUNITY | 282 | 4 | 7.00E-04 | 2.39E-02 | IL1B, HLA-G, IL18, PVRL2 |
| GOBP CELLULAR HOMEOSTASIS | 766 | 6 | 7.14E-04 | 2.43E-02 | HTR1B, CACNB2, DRD4, CACNA1C, ACE, TMPRSS6 |
| **GOBP MAPK CASCADE** | **768** | **6** | **7.24E-04** | **2.45E-02** | **IL1B, CTNNB1, FGA, HMGCR, DRD4, IL18** |
| GOBP EMBRYONIC SKELETAL SYSTEM DEVELOPMENT | 123 | 3 | 7.36E-04 | 2.48E-02 | TGFBR2, CTNNB1, SIX1 |
| GOBP REGULATION OF MULTICELLULAR ORGANISMAL DEVELOPMENT | 1417 | 8 | 7.49E-04 | 2.51E-02 | IL1B, TGFBR2, CTNNB1, HLA-G, ZNF365, IL18, SIX1, ACE |
| GOBP REGULATION OF ANIMAL ORGAN FORMATION | 29 | 2 | 7.98E-04 | 2.66E-02 | CTNNB1, SIX1 |
| GOBP EMBRYONIC FORELIMB MORPHOGENESIS | 29 | 2 | 7.98E-04 | 2.66E-02 | CTNNB1, CACNA1C |
| GOBP REGULATION OF ESTABLISHMENT OF PROTEIN LOCALIZATION | 517 | 5 | 8.19E-04 | 2.71E-02 | IL1B, FGA, HMGCR, TCF7L2, DRD4 |
| GOBP POSITIVE REGULATION OF LYMPHOCYTE DIFFERENTIATION | 128 | 3 | 8.26E-04 | 2.72E-02 | TGFBR2, HLA-G, IL18 |
| GOBP REGULATION OF PROTEIN MODIFICATION PROCESS | 1441 | 8 | 8.36E-04 | 2.75E-02 | IL1B, TGFBR2, CTNNB1, HMGCR, AUTS2, DRD4, IL18, ACE |
| GOBP NEGATIVE REGULATION OF ESTABLISHMENT OF PROTEIN LOCALIZATION | 129 | 3 | 8.45E-04 | 2.76E-02 | IL1B, HMGCR, DRD4 |
| GOBP TOLERANCE INDUCTION | 30 | 2 | 8.55E-04 | 2.76E-02 | TGFBR2, HLA-G |
| GOBP REGULATION OF T HELPER 1 TYPE IMMUNE RESPONSE | 30 | 2 | 8.55E-04 | 2.76E-02 | IL1B, IL18 |
| GOBP POSITIVE REGULATION OF VASOCONSTRICTION | 30 | 2 | 8.55E-04 | 2.76E-02 | FGA, ACE |
| GOBP ACTIN FILAMENT BASED MOVEMENT | 130 | 3 | 8.64E-04 | 2.77E-02 | SCN5A, CACNB2, CACNA1C |
| GOBP NEGATIVE REGULATION OF MONOATOMIC ION TRANSPORT | 130 | 3 | 8.64E-04 | 2.77E-02 | HTR1B, DRD4, ACE |
| GOBP POSITIVE REGULATION OF PRODUCTION OF MOLECULAR MEDIATOR OF IMMUNE RESPONSE | 131 | 3 | 8.83E-04 | 2.81E-02 | IL1B, HLA-G, IL18 |
| GOBP CARDIAC MUSCLE CONTRACTION | 131 | 3 | 8.83E-04 | 2.81E-02 | SCN5A, CACNB2, CACNA1C |
| GOBP HEAD DEVELOPMENT | 799 | 6 | 8.90E-04 | 2.82E-02 | TMEM57, TGFBR2, SCN5A, CTNNB1, ZNF365, ACE |
| GOBP ARACHIDONIC ACID SECRETION | 31 | 2 | 9.13E-04 | 2.88E-02 | DRD4, ACE |
| GOBP LEUKOCYTE MEDIATED CYTOTOXICITY | 133 | 3 | 9.23E-04 | 2.90E-02 | HLA-G, IL18, PVRL2 |
| GOBP T CELL ACTIVATION | 532 | 5 | 9.31E-04 | 2.91E-02 | IL1B, TGFBR2, CTNNB1, HLA-G, IL18 |
| GOBP NEGATIVE REGULATION OF NUCLEOBASE CONTAINING COMPOUND METABOLIC PROCESS | 1468 | 8 | 9.44E-04 | 2.94E-02 | PRDM16, CTNNB1, BACH2, ESR1, GLIS3, TCF7L2, SIX1, TMPRSS6 |
| **GOBP REGULATION OF CALCIUM ION IMPORT** | **32** | **2** | **9.73E-04** | **2.98E-02** | **CTNNB1, ACE** |
| GOBP POSITIVE REGULATION OF SODIUM ION TRANSPORT | 32 | 2 | 9.73E-04 | 2.98E-02 | SCN5A, DRD4 |
| GOBP POSITIVE REGULATION OF NATURAL KILLER CELL MEDIATED IMMUNITY | 32 | 2 | 9.73E-04 | 2.98E-02 | HLA-G, PVRL2 |
| GOBP NEGATIVE REGULATION OF CELL JUNCTION ASSEMBLY | 32 | 2 | 9.73E-04 | 2.98E-02 | IL1B, ACE |
| GOBP APOPTOTIC PROCESS | 1858 | 9 | 9.96E-04 | 3.04E-02 | IL1B, TGFBR2, CTNNB1, FGA, HLA-G, ESR1, TCF7L2, SIX1, ACE |
| GOBP VESICLE MEDIATED TRANSPORT | 1486 | 8 | 1.02E-03 | 3.11E-02 | IL1B, TGFBR2, CTNNB1, FGA, TIMD4, HTR1B, DRD4, STARD3 |
| GOBP HEART FORMATION | 33 | 2 | 1.03E-03 | 3.11E-02 | CTNNB1, SIX1 |
| GOBP HEPARAN SULFATE PROTEOGLYCAN BIOSYNTHETIC PROCESS | 33 | 2 | 1.03E-03 | 3.11E-02 | CTNNB1, TCF7L2 |
| GOBP MYOBLAST PROLIFERATION | 33 | 2 | 1.03E-03 | 3.11E-02 | CTNNB1, SIX1 |
| GOBP GLIOGENESIS | 314 | 4 | 1.04E-03 | 3.11E-02 | IL1B, CTNNB1, PRDM8, ZNF365 |
| GOBP REGULATION OF LEUKOCYTE DIFFERENTIATION | 314 | 4 | 1.04E-03 | 3.11E-02 | TGFBR2, CTNNB1, HLA-G, IL18 |
| GOBP REGULATION OF TUBE SIZE | 139 | 3 | 1.05E-03 | 3.11E-02 | FGA, HTR1B, ACE |
| GOBP POSITIVE REGULATION OF LYMPHOCYTE ACTIVATION | 316 | 4 | 1.07E-03 | 3.15E-02 | IL1B, TGFBR2, HLA-G, IL18 |
| GOBP ACTION POTENTIAL | 140 | 3 | 1.07E-03 | 3.15E-02 | SCN5A, CACNB2, CACNA1C |
| **GOBP CALCIUM ION HOMEOSTASIS** | **318** | **4** | **1.09E-03** | **3.19E-02** | **HTR1B, CACNB2, DRD4, CACNA1C** |
| GOBP PRESYNAPTIC MODULATION OF CHEMICAL SYNAPTIC TRANSMISSION | 34 | 2 | 1.10E-03 | 3.19E-02 | HTR1B, CHRNA5 |
| GOBP POSITIVE REGULATION OF ANIMAL ORGAN MORPHOGENESIS | 34 | 2 | 1.10E-03 | 3.19E-02 | CTNNB1, SIX1 |
| GOBP DEVELOPMENTAL INDUCTION | 34 | 2 | 1.10E-03 | 3.19E-02 | CTNNB1, SIX1 |
| GOBP NEGATIVE REGULATION OF TRANSMEMBRANE TRANSPORT | 143 | 3 | 1.14E-03 | 3.29E-02 | IL1B, DRD4, ACE |
| GOBP VENTRICULAR CARDIAC MUSCLE CELL ACTION POTENTIAL | 35 | 2 | 1.16E-03 | 3.34E-02 | SCN5A, CACNA1C |
| GOBP ANTIGEN PROCESSING AND PRESENTATION OF PEPTIDE ANTIGEN VIA MHC CLASS I | 35 | 2 | 1.16E-03 | 3.34E-02 | HLA-G, ACE |
| GOBP RESPONSE TO STEROID HORMONE | 324 | 4 | 1.17E-03 | 3.35E-02 | TGFBR2, HTR1B, ESR1, ACE |
| GOBP EPITHELIAL TUBE MORPHOGENESIS | 326 | 4 | 1.20E-03 | 3.42E-02 | TGFBR2, CTNNB1, ESR1, SIX1 |
| GOBP REGULATION OF CELLULAR RESPONSE TO GROWTH FACTOR STIMULUS | 327 | 4 | 1.21E-03 | 3.44E-02 | PRDM16, IL1B, CTNNB1, TMPRSS6 |
| GOBP BRANCHING INVOLVED IN BLOOD VESSEL MORPHOGENESIS | 36 | 2 | 1.23E-03 | 3.46E-02 | TGFBR2, CTNNB1 |
| GOBP POSITIVE REGULATION OF MORPHOGENESIS OF AN EPITHELIUM | 36 | 2 | 1.23E-03 | 3.46E-02 | CTNNB1, SIX1 |
| GOBP AORTA MORPHOGENESIS | 36 | 2 | 1.23E-03 | 3.46E-02 | TGFBR2, SIX1 |
| GOBP POSITIVE REGULATION OF INFLAMMATORY RESPONSE | 148 | 3 | 1.26E-03 | 3.51E-02 | IL1B, IL18, ACE |
| GOBP REGULATION OF EXTRINSIC APOPTOTIC SIGNALING PATHWAY | 149 | 3 | 1.28E-03 | 3.56E-02 | IL1B, FGA, TCF7L2 |
| GOBP POSITIVE REGULATION OF HISTONE METHYLATION | 37 | 2 | 1.30E-03 | 3.56E-02 | CTNNB1, AUTS2 |
| GOBP SEROTONIN RECEPTOR SIGNALING PATHWAY | 37 | 2 | 1.30E-03 | 3.56E-02 | HTR1B, DRD4 |
| GOBP RESPONSE TO MINERALOCORTICOID | 37 | 2 | 1.30E-03 | 3.56E-02 | HTR1B, ACE |
| GOBP SPECIFICATION OF ANIMAL ORGAN IDENTITY | 37 | 2 | 1.30E-03 | 3.56E-02 | CTNNB1, SIX1 |
| GOBP FORELIMB MORPHOGENESIS | 37 | 2 | 1.30E-03 | 3.56E-02 | CTNNB1, CACNA1C |
| GOBP REGULATION OF MONOATOMIC CATION TRANSMEMBRANE TRANSPORT | 334 | 4 | 1.31E-03 | 3.58E-02 | SCN5A, CACNB2, DRD4, CACNA1C |
| GOBP HINDBRAIN DEVELOPMENT | 151 | 3 | 1.33E-03 | 3.62E-02 | SCN5A, CTNNB1, ZNF365 |
| GOBP REGULATION OF VASCULATURE DEVELOPMENT | 337 | 4 | 1.35E-03 | 3.67E-02 | IL1B, TGFBR2, CTNNB1, HLA-G |
| GOBP T CELL CYTOKINE PRODUCTION | 38 | 2 | 1.37E-03 | 3.69E-02 | IL1B, IL18 |
| GOBP POSITIVE REGULATION OF T CELL MEDIATED CYTOTOXICITY | 38 | 2 | 1.37E-03 | 3.69E-02 | HLA-G, PVRL2 |
| GOBP EXPORT FROM CELL | 870 | 6 | 1.38E-03 | 3.70E-02 | IL1B, FGA, HMGCR, HTR1B, TCF7L2, DRD4 |
| GOBP ORGANIC ACID TRANSPORT | 339 | 4 | 1.38E-03 | 3.70E-02 | IL1B, HTR1B, DRD4, ACE |
| GOBP METHYLATION | 342 | 4 | 1.43E-03 | 3.81E-02 | PRDM16, CTNNB1, PRDM8, AUTS2 |
| GOBP CELL CELL JUNCTION ASSEMBLY | 155 | 3 | 1.43E-03 | 3.81E-02 | IL1B, CTNNB1, ACE |
| GOBP NEGATIVE REGULATION OF MULTICELLULAR ORGANISMAL PROCESS | 1208 | 7 | 1.46E-03 | 3.85E-02 | IL1B, TGFBR2, CTNNB1, FGA, HMGCR, HLA-G, ACE |
| GOBP POSITIVE REGULATION OF TRANSPORT | 882 | 6 | 1.48E-03 | 3.90E-02 | IL1B, SCN5A, FGA, CACNB2, TCF7L2, DRD4 |
| GOBP LEUKOCYTE DIFFERENTIATION | 591 | 5 | 1.48E-03 | 3.90E-02 | IL1B, TGFBR2, CTNNB1, HLA-G, IL18 |
| GOBP REGULATION OF NEUROINFLAMMATORY RESPONSE | 40 | 2 | 1.52E-03 | 3.96E-02 | IL1B, IL18 |
| GOBP REGULATION OF ACTIN FILAMENT BASED MOVEMENT | 40 | 2 | 1.52E-03 | 3.96E-02 | SCN5A, CACNA1C |
| **GOBP CALCIUM ION TRANSMEMBRANE TRANSPORT** | **348** | **4** | **1.52E-03** | **3.96E-02** | **SCN5A, CACNB2, DRD4, CACNA1C** |
| GOBP GROWTH | 895 | 6 | 1.60E-03 | 4.13E-02 | TGFBR2, CTNNB1, ESR1, AUTS2, SIX1, FTO |
| GOBP MALE SEX DIFFERENTIATION | 161 | 3 | 1.60E-03 | 4.13E-02 | CTNNB1, ESR1, ACE |
| GOBP HEMOPOIESIS | 896 | 6 | 1.60E-03 | 4.13E-02 | IL1B, TGFBR2, CTNNB1, HLA-G, IL18, ACE |
| GOBP REGULATION OF ANATOMICAL STRUCTURE MORPHOGENESIS | 899 | 6 | 1.63E-03 | 4.19E-02 | IL1B, TGFBR2, CTNNB1, HLA-G, ESR1, SIX1 |
| GOBP REGULATION OF OLIGODENDROCYTE DIFFERENTIATION | 42 | 2 | 1.67E-03 | 4.27E-02 | CTNNB1, ZNF365 |
| GOBP POSITIVE REGULATION OF CD4 POSITIVE ALPHA BETA T CELL ACTIVATION | 42 | 2 | 1.67E-03 | 4.27E-02 | TGFBR2, IL18 |
| GOBP REGULATION OF CELL DEATH | 1609 | 8 | 1.70E-03 | 4.33E-02 | IL1B, CTNNB1, FGA, HLA-G, ESR1, TCF7L2, SIX1, ACE |
| GOBP POSITIVE REGULATION OF LEUKOCYTE PROLIFERATION | 165 | 3 | 1.71E-03 | 4.34E-02 | IL1B, TGFBR2, IL18 |
| GOBP IMMUNE EFFECTOR PROCESS | 611 | 5 | 1.72E-03 | 4.34E-02 | IL1B, HLA-G, IL18, ACE, PVRL2 |
| GOBP T HELPER 1 TYPE IMMUNE RESPONSE | 43 | 2 | 1.75E-03 | 4.40E-02 | IL1B, IL18 |
| GOBP MAMMARY GLAND MORPHOGENESIS | 43 | 2 | 1.75E-03 | 4.40E-02 | TGFBR2, ESR1 |
| GOBP REGULATION OF APOPTOTIC SIGNALING PATHWAY | 362 | 4 | 1.76E-03 | 4.40E-02 | IL1B, CTNNB1, FGA, TCF7L2 |
| GOBP EMBRYONIC CRANIAL SKELETON MORPHOGENESIS | 44 | 2 | 1.84E-03 | 4.55E-02 | TGFBR2, SIX1 |
| GOBP AUTONOMIC NERVOUS SYSTEM DEVELOPMENT | 44 | 2 | 1.84E-03 | 4.55E-02 | CTNNB1, SIX1 |
| GOBP POSITIVE REGULATION OF BINDING | 169 | 3 | 1.84E-03 | 4.55E-02 | CTNNB1, TCF7L2, ACE |
| GOBP MONOCARBOXYLIC ACID TRANSPORT | 171 | 3 | 1.90E-03 | 4.69E-02 | IL1B, DRD4, ACE |
| **GOBP REGULATION OF VOLTAGE GATED CALCIUM CHANNEL ACTIVITY** | **45** | **2** | **1.92E-03** | **4.69E-02** | **CACNB2, DRD4** |
| GOBP RESPONSE TO COCAINE | 45 | 2 | 1.92E-03 | 4.69E-02 | HTR1B, DRD4 |
| GOBP GENITALIA DEVELOPMENT | 45 | 2 | 1.92E-03 | 4.69E-02 | CTNNB1, ESR1 |
| GOBP REGULATION OF IMMUNE EFFECTOR PROCESS | 371 | 4 | 1.93E-03 | 4.69E-02 | IL1B, HLA-G, IL18, PVRL2 |
| GOBP REGULATION OF NEUROGENESIS | 373 | 4 | 1.96E-03 | 4.76E-02 | IL1B, CTNNB1, ZNF365, ACE |
| GOBP BEHAVIOR | 630 | 5 | 1.96E-03 | 4.76E-02 | HMGCR, HTR1B, DRD4, CHRNA5, ACE |
| GOBP DENDRITIC CELL DIFFERENTIATION | 46 | 2 | 2.00E-03 | 4.84E-02 | TGFBR2, HLA-G |
| GOBP STRIATED MUSCLE CONTRACTION | 175 | 3 | 2.03E-03 | 4.88E-02 | SCN5A, CACNB2, CACNA1C |
| GOBP REGULATION OF T CELL DIFFERENTIATION | 176 | 3 | 2.06E-03 | 4.95E-02 | TGFBR2, HLA-G, IL18 |
| GOBP ALPHA BETA T CELL PROLIFERATION | 47 | 2 | 2.09E-03 | 4.97E-02 | TGFBR2, IL18 |
| GOBP PROSTATE GLAND DEVELOPMENT | 47 | 2 | 2.09E-03 | 4.97E-02 | CTNNB1, ESR1 |
| GOBP REGULATION OF ENDOTHELIAL CELL DIFFERENTIATION | 47 | 2 | 2.09E-03 | 4.97E-02 | IL1B, CTNNB1 |

**Supplementary Table 7:** Combination of genes and their effect on various phenotypes.

| **GeneSet** | **N** | **n** | **P-value** | **adjusted P** | **genes** |
| --- | --- | --- | --- | --- | --- |
| **Systolic blood pressure** | **704** | **11** | **2.47E-09** | **1.09E-05** | **PRDM16, TGFBR2, CTNNB1, PRDM8, ESR1, GLIS3, CACNB2, ZNF365, TCF7L2, FTO, ACE** |
| Medication use (HMG CoA reductase inhibitors) | 64 | 5 | 3.30E-08 | 7.30E-05 | HMGCR, TIMD4, NAT2, TCF7L2, FTO |
| Medication use (agents acting on the renin-angiotensin system) | 113 | 5 | 5.77E-07 | 7.41E-04 | PRDM16, CACNB2, CACNA1C, FTO, ACE |
| **LDL cholesterol levels** | **220** | **6** | **6.70E-07** | **7.41E-04** | **HMGCR, TIMD4, HLA-G, NAT2, CACNB2, FTO** |
| **Diastolic blood pressure** | **555** | **8** | **9.61E-07** | **8.51E-04** | **PRDM16, PRDM8, ESR1, AUTS2, CACNB2, ZNF365, CACNA1C, ACE** |
| Medication use (calcium channel blockers) | 71 | 4 | 3.27E-06 | 2.41E-03 | NAT2, CACNB2, FTO, ACE |
| **LDL cholesterol levels x long total sleep time interaction (2df test)** | **24** | **3** | **5.42E-06** | **3.04E-03** | **TMEM57, HMGCR, TIMD4** |
| Breast cancer (estrogen-receptor negative) | 25 | 3 | 6.16E-06 | 3.04E-03 | ESR1, ZNF365, FTO |
| Breast cancer | 184 | 5 | 6.36E-06 | 3.04E-03 | TGFBR2, ESR1, ZNF365, TCF7L2, FTO |
| Red blood cell count | 509 | 7 | 6.86E-06 | 3.04E-03 | PRDM16, PRDM8, BACH2, AUTS2, GLIS3, CACNA1C, TMPRSS6 |
| **Apolipoprotein B levels** | **203** | **5** | **1.02E-05** | **4.12E-03** | **HMGCR, TIMD4, HLA-G, NAT2, CACNB2** |
| Clinical laboratory measurements | 6 | 2 | 3.01E-05 | 1.11E-02 | TCF7L2, TMPRSS6 |
| Exploratory eye movement dysfunction in schizophrenia (cognitive search score) | 7 | 2 | 4.21E-05 | 1.24E-02 | PRDM8, CACNA1C |
| Breast Cancer in BRCA1 mutation carriers | 7 | 2 | 4.21E-05 | 1.24E-02 | ESR1, TCF7L2 |
| Exhaled carbon monoxide levels in smokers with chronic obstructive pulmonary disease | 7 | 2 | 4.21E-05 | 1.24E-02 | SIX1, CHRNA5 |
| Iron status biomarkers (transferrin levels) | 8 | 2 | 5.61E-05 | 1.55E-02 | NAT2, TMPRSS6 |
| Breast size | 59 | 3 | 8.42E-05 | 2.19E-02 | ESR1, ZNF365, FTO |
| Mammographic density (dense area) | 11 | 2 | 1.10E-04 | 2.63E-02 | ESR1, ZNF365 |
| Hemoglobin | 339 | 5 | 1.18E-04 | 2.63E-02 | PRDM16, GLIS3, TCF7L2, CACNA1C, TMPRSS6 |
| **Triglyceride levels** | **548** | **6** | **1.19E-04** | **2.63E-02** | **TIMD4, HLA-G, NAT2, FTO, STARD3, TMPRSS6** |
| **Circulating leptin levels or type 2 diabetes** | **12** | **2** | **1.32E-04** | **2.65E-02** | **GLIS3, TCF7L2** |
| **Fasting insulin** | **69** | **3** | **1.34E-04** | **2.65E-02** | **GLIS3, TCF7L2, FTO** |
| **Type 2 diabetes** | **563** | **6** | **1.38E-04** | **2.65E-02** | **HMGCR, AUTS2, GLIS3, TCF7L2, FTO, ACE** |
| Alcohol consumption | 71 | 3 | 1.46E-04 | 2.70E-02 | AUTS2, FTO, STARD3 |
| Systolic blood pressure x alcohol consumption interaction (2df test) | 73 | 3 | 1.59E-04 | 2.78E-02 | CACNB2, FTO, ACE |
| **Total cholesterol levels** | **192** | **4** | **1.63E-04** | **2.78E-02** | **HMGCR, TIMD4, NAT2, GLIS3** |
| Mosquito bite size | 83 | 3 | 2.32E-04 | 3.77E-02 | BACH2, AUTS2, GNG11 |
| **Blood glucose levels** | **16** | **2** | **2.39E-04** | **3.77E-02** | **TCF7L2, FTO** |
